# Lie symmetry approach to the dynamical behavior and conservation laws of actin filament electrical models

**DOI:** 10.1371/journal.pone.0331243

**Published:** 2025-09-09

**Authors:** Beenish, Maria Samreen, Fehaid Salem Alshammari

**Affiliations:** 1 Department of Mathematics, Quaid-I-Azam University, Islamabad, Pakistan; 2 Department of Mathematics and Statistics, College of Science, Imam Mohammad Ibn Saud Islamic University (IMSIU), Riyadh, Saudi Arabia; Federal University of Technology - Parana, BRAZIL

## Abstract

This research explores the dynamical properties and solutions of actin filaments, which serve as electrical conduits for ion transport along their lengths. Utilizing the Lie symmetry approach, we identify symmetry reductions that simplify the governing equation by lowering its dimensionality. This process leads to the formulation of a second-order differential equation, which, upon applying a Galilean transformation, is further converted into a system of first-order differential equations. Additionally, we investigate the bifurcation structure and sensitivity of the proposed dynamical system. When subjected to an external force, the system exhibits quasi-periodic behavior, which is detected using chaos analysis tools. Sensitivity analysis is also performed on the unperturbed system under varying initial conditions. Moreover, we establish the conservation laws associated with the equation and conduct a stability analysis of the model. Employing the tanh method, we derive exact solutions and visualize them through 3D and 2D graphical representations to gain deeper insights. These findings offer new perspectives on the studied equation and significantly contribute to the understanding of nonlinear wave dynamics.

## 1 Introduction

**Fig 1:**
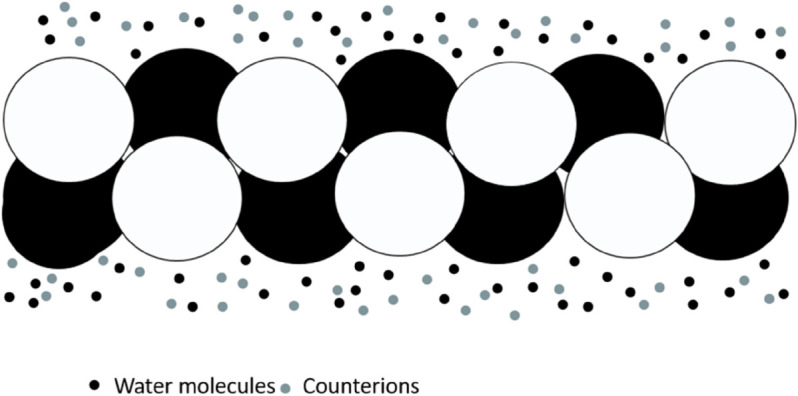
F-actin is enveloped by water molecules and counterions.

**Fig 2:**
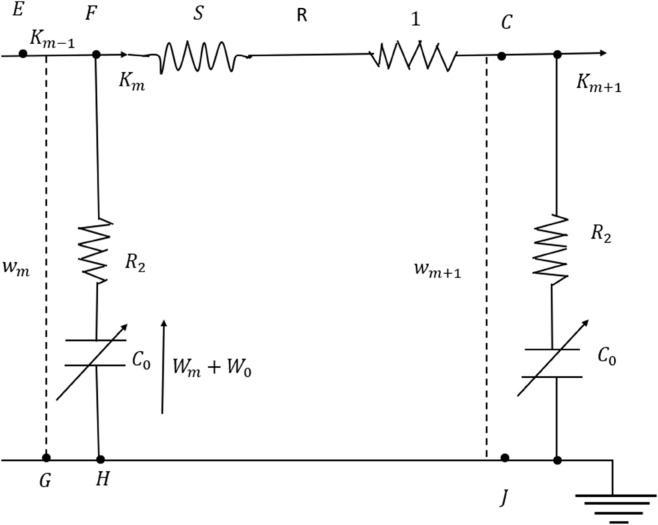
A simplified circuit for the ${\text{m}}^{\text{th}}$ monomer shows current $J_{m}$ flowing through inductance S and resistance ${ℜ}_{1}$.

The study of NLPDEs and their corresponding solutions has increasingly drawn focus as a pivotal and demanding area in both pure and applied mathematics. These equations play a crucial role in understanding nonlinear physical phenomena, with applications spanning engineering and natural sciences. Exact or analytical solutions are highly valued for their ability to reveal the intrinsic properties of nonlinear systems [1–4]. Given their broad relevance in nonlinear sciences, interest in studying NLPDEs has grown substantially. Various mathematical techniques, including the modified generalized exponential rational function method [5], the unifed method [6], the group analysis [7,8], the Lie isomorphism method [9], have been employed to obtain solitary wave solutions [10–12]. The cytoskeleton is an essential component of all living cells, consisting of three filamentous structures: actin-based microfilaments, intermediate filaments, and tubulin-based microtubules. These networks, interconnected by specific proteins, regulate cellular processes such as migration, division, and intracellular transport. actin-based microfilaments facilitate cell migration and remodeling at the leading edge, while tubulin-based microtubules anchor chromosomes and mediate cell division. Additionally, molecular motors, as protein complexes, facilitate directional transport along tubulin-based microtubules and actin-based microfilaments [13,14]. In the analysis of F-actin surrounded by a saline solution see Fig 1, it is important to determine the resistance, inductance, and capacitance of the entire filament. This requires obtaining effective values using the appropriate properties of addition. As shown in Fig 2, both parallel and series components contribute to the total resistance, the capacitance follows a parallel-addition property, and the inductance is strictly a series contribution. For a filament consisting of *m* monomers, the effective resistance (R), inductance (*S*), and capacitance (C) are given by [15]:

$$\left\{ \begin{array}{c} R_{eff}=(\sum_{p=1}^{m}\frac{1}{R_{2,p}}{)}^{-1}+\sum_{p=1}^{m}R_{1,p},\\ S_{eff}=\sum_{p=1}^{m}S_{p},\;\;\;C_{eff}=\sum_{p=1}^{m}C_{0,p}. \end{array}\right.$$ (1)

Where $R_{1,p}=6.11\times 10^{6}\;\Omega$ and $R_{2,p}=0.9\times 10^{6}\;\Omega$ , satisfying the relation $R_{1,p}=7R_{2,p}$. It is important to note that we have assigned $R_{1,p}=R_{1}$, $R_{2,p}=R_{2}$, *S*_*p*_ = *S*, and $C_{0,p}=C_{0}$. Consequently, for a 1μm segment of the actin filament, we obtain $R_{eff}=1.2\times 10^{9}\;\Omega ,\;\;S_{eff}=340\times 10^{-12}\;H,\;\;\Omega ,\;\;C_{eff}=0.02\times 10^{-12}\;F.$

In this part, we establish an electrical model of the actin filament using inductive, capacitive, and resistive components, building on the concepts outlined earlier. Essentially, Kirchhoff’s laws are applied to the segment of the effective electrical circuit corresponding to a single monomer, *N*, which is coupled to neighboring monomers see Fig 2. These Bjerrum ions have been shown to generate a time-dependent current as they move along helical paths, contributing to the inductance denoted as *S*. Due to viscosity, a resistive component is also anticipated in these currents, which is included in series with *S* and labeled as $R_{1}$ see Fig 2. In parallel with these components, another resistance [16,17], referred to as $R_{2}$, exists between the Bjerrum ions and the filament surface. A capacitance $C_{0}$ is placed in series with this resistance, and it is assumed that the charge on this capacitor exhibits a nonlinear relationship with voltage, similar to the charge-voltage behavior of a reverse-biased pn-junction diode as described by Ma *et al*. [1]. Consequently, for the *mth* monomer, we assume [18,19]:

$$V_{m}=C_{0}(W_{0}-\alpha W_{m}^{2}),$$ (2)

where *α* is anticipated to be small. Applying Kirchhoff’s laws and referring to Fig 2, if *K*_*m*_ represents the current passing through the inductance *S* and resistor $R_{1}$, while *K*_*m*−1_ is the current flowing along EF, then the current through HF must be $K_{m}-K_{m-1}$. For the BC segment of the *mth* monomer:

$$w_{m}-w_{m+1}=S\frac{dK_{m}}{d\tau }+K_{m}R_{1},$$ (3)

where *w*_*m*_ and *w*_*m* + 1_ represent the voltages across EG and CJ, respectively, as illustrated in Fig 2. Likewise, if the voltage across the capacitor is *W*_*m*_ − *W*_0_, where *W*_0_ denotes the capacitor’s bias voltage, then we obtain:

$$w_{m}=R_{2}(K_{m-1}-K_{m})+W_{0}+W_{m}.$$ (4)

The current through section FH equals the rate of change of charge $V_{m}$, giving:

$$K_{m-1}-K_{m}=\frac{dV_{m}}{d\tau }\cdot$$ (5)

From Eq (3), it follows that:

$$S\frac{dV_{m-1}}{d\tau }=w_{m-1}-w_{m}-K_{m-1}R_{1},$$ (6)

and

$$S\frac{dV_{m}}{d\tau }=w_{m}-w_{m+1}-K_{m}R_{1}.$$ (7)

From Eq (5), it follows that:

$$S\frac{d^{2}V_{m}}{d\tau^{2}}=S\frac{dK_{m-1}}{d\tau }-S\frac{dK_{m}}{d\tau }.$$ (8)

Thus, using Eqs (6)–(8), we obtain:

$$S\frac{d^{2}V_{m}}{d\tau^{2}}=w_{m-1}-w_{m}-K_{m-1}R_{1}-(W_{m}-W_{m+1}-K_{m}R_{1}),\;=W_{m+1}+W_{m-1}-2W_{m}+R_{1}(K_{m}-K_{m+1}).$$ (9)

Substituting Eq (4) into Eq (9) gives:

$$S\frac{d^{2}V_{m}}{d\tau^{2}}=SC_{0}(W_{m}-\alpha W_{m}^{2})\;=W_{m+1}+W_{m-1}-2W_{m}-R_{1}C_{0}\frac{d}{d\tau }(W_{m}-\alpha W_{m}^{2})-R_{2}C_{0}\{2\frac{d}{d\tau }(W_{m}-\alpha W_{m}^{2})\;-\frac{d}{d\tau }(W_{m+1}-\alpha W_{m+1}^{2})-\frac{d}{d\tau }(W_{m-1}-\alpha W_{m-1}^{2})\}.$$ (10)

Applying a continuum approximation with *W*_*m*_ = *W* and a Taylor expansion in *β* gives:

$$W_{m+1}=W+\beta (\frac{\partial W}{\partial \vartheta })+\frac{\beta^{2}}{2!}(\frac{\partial^{2}W}{\partial \vartheta^{2}})+\frac{\beta^{3}}{3!}(\frac{\partial^{3}W}{\partial \vartheta^{3}})+\frac{\beta^{4}}{4!}(\frac{\partial^{4}W}{\partial \vartheta^{4}})+......\;W_{m-1}=W-\beta (\frac{\partial W}{\partial \vartheta })+\frac{\beta^{2}}{2!}(\frac{\partial^{2}W}{\partial \vartheta^{2}})-\frac{\beta^{3}}{3!}(\frac{\partial^{3}W}{\partial \vartheta^{3}})+\frac{\beta^{4}}{4!}(\frac{\partial^{4}W}{\partial \vartheta^{4}})+......$$ (11)

At this point, all derivatives up to the fourth order are considered. Using Eq (11), we get:

$$W_{m+1}-2W_{m}+W_{m-1}=\beta^{2}(\frac{\partial^{2}W}{\partial \vartheta^{2}})+\frac{2\beta^{4}}{4!}(\frac{\partial^{4}W}{\partial \vartheta^{4}}).$$ (12)

Applying Eq (12), and Eq (11), we rewrite Eq (10) as:

$$SC_{0}\frac{\partial }{\partial \tau }(W-\alpha W^{2})=\beta \frac{\partial^{2}W}{\partial \vartheta^{2}}+\frac{2\beta^{4}}{4!}\frac{\partial^{3}W}{\partial \vartheta^{3}}-R_{1}C_{0}\frac{\partial }{\partial \tau }(W-\alpha W^{2})+R_{2}C_{0}\frac{\partial }{\partial \tau }[\beta^{2}\frac{\partial^{2}W}{\partial \vartheta^{2}}\;+\frac{2\beta^{4}}{4!}\frac{\partial^{4}W}{\partial \vartheta^{4}}]-R_{2}C_{0}\alpha \frac{\partial }{\partial \tau }[2\beta^{2}\frac{\partial^{2}W}{\partial \vartheta^{2}}+2\beta^{2}(\frac{\partial W}{\partial \vartheta }{)}^{2}+\frac{\beta^{4}W}{6}\frac{\partial^{4}W}{\partial \vartheta^{4}}+\frac{2\beta^{4}}{3}(\frac{\partial W}{\partial \vartheta })(\frac{\partial^{3}W}{\partial \vartheta^{3}})\;+\frac{\beta^{4}}{2}(\frac{\partial^{2}W}{\partial \vartheta^{2}}{)}^{2}].$$ (13)

Assuming time variations are small relative to the constant background voltage, we consider the nonlinear capacitance term as second order. With time derivatives of order *ε*, nonlinear voltage terms of order $\epsilon^{2}$, and *β* of order *ε*, we retain terms up to $\epsilon^{3}$ in Eq (13) to derive the final expression [20]:

$$-SC_{0}\frac{\partial^{2}W}{\partial \tau^{2}}+\beta^{2}\frac{\partial^{2}W}{\partial \vartheta^{2}}+R_{2}C_{0}\frac{\partial }{\partial \tau }(\beta^{2}\frac{\partial^{2}W}{\partial \vartheta^{2}})-R_{1}C_{0}\frac{\partial W}{\partial \tau }+2\alpha R_{1}C_{0}W\frac{\partial W}{\partial \tau }=0.$$ (14)

Eq (14) presents *W* as the dependent variable, while *τ* and ϑ are the independent variables. Specifically, *τ* corresponds to the temporal variable, and ϑ denotes the spatial variable. J. A. Tuszynski *et al*. [20] studied Eq (14), solving it using the maximum propagation velocity wave expressed in Jacobi elliptic functions. In this work, we analyze Eq (14) from different perspectives: Firstly, we conduct a symmetry analysis of Eq (14), derive its symmetry group, and obtain solutions via the tanh method. Additionally, we visualize the solution’s behavior using Mathematica. Secondly, we examine the dynamical behavior through bifurcation and chaos analysis. Thirdly, we investigate chaotic behavior using methods such as time series analysis, poincare maps, power spectrum, return maps, fractal dimension, and Lyapunov exponents. Each tool has distinct significance in chaos detection:

- **Time analysis:** Investigates the progression of a system’s state variables across temporal dimensions, discerning patterns, trends, and anomalies that signify chaotic behavior.
- **Poincare map:** Represents a discrete framework of a continuous dynamical system, encapsulating its intersections with a lower-dimensional subspace, thereby elucidating periodicity or chaotic trajectories.
- **Power spectrum:** Examines the frequency constituents of a time series to differentiate between regular and chaotic dynamics, chaotic systems manifest broad, continuous spectra as opposed to distinct peaks.
- **Return map:** Illustrates the correlation between consecutive values of a variable, facilitating the visualization of attractors, periodicity, and the manifestation of chaos through complex structures.
- **Lyapunov exponent:** Quantifies the sensitivity of trajectories to initial conditions, with positive values denoting exponential divergence and affirming chaotic behavior.
- **Fractal dimension:** The fractal dimension quantifies the complexity of chaotic attractors, reflecting the scaling behavior of system intricacies. A non-integer value indicates self-similarity and irregular dynamics.
- Lastly, we highlight three further aspects of our study. we perform a sensitivity analysis of Eq (14). We investigate the conservation laws of Eq (14). We analyze the stability of Eq (14).

**Fig 3:**
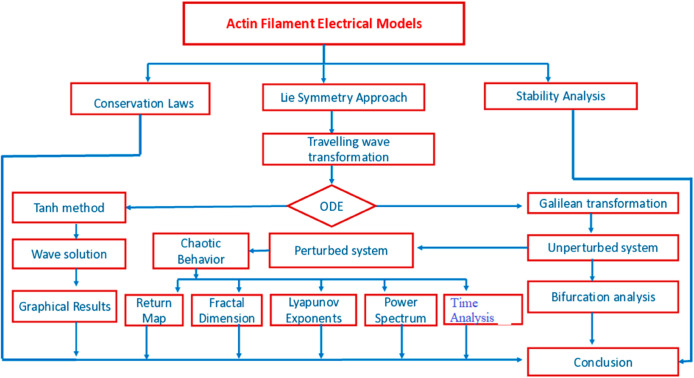
Chart of the implemented analysis.

Lie point symmetry plays a crucial role in various scientific fields, especially in integrable systems with infinitely many symmetries. The Lie symmetry analysis method [21] is recognized as an effective approach for obtaining analytical solutions to NLPDEs. Additionally, it is instrumental in deriving conservation laws, which are essential for studying nonlinear physical phenomena [22]. A significant recent contribution to nonlinear physics can be found in [23]. Conservation laws mathematically represent the principle that a specific physical quantity remains constant during the evolution of a physical system. They also aid in refining mathematical methods to establish the existence and uniqueness of solutions. Various techniques are available for formulating conservation laws for differential equations (DEs) [24,25]. The well-known Noether theorem [26] links Lie point symmetries to conservation laws, while Ibragimov [27] introduced a new conservation law theorem. Conservation laws, symmetry analysis, bifurcation, and chaos analysis have become prominent research topics in recent studies. Tariq Mahmood *et al*. [28] investigated symmetry analysis, conservation laws, and bifurcation analysis using the Klein–Gordon equation. The graphical abstract has been depicted in Fig 3.

## 2 Lie point symmetry analysis

A one-parameter Lie group of infinitesimal transformations is presented [29,30]:

$$\Xi =\Theta_{a}(\vartheta ,\tau ,W)\frac{\partial }{\partial \vartheta }+\Theta_{b}(\vartheta ,\tau ,W)\frac{\partial }{\partial \tau }+\mu (\vartheta ,\tau ,W)\frac{\partial }{\partial W}.$$ (15)

Here, $\Theta_{a}$, $\Theta_{b}$, and μ are infinitesimal generators, and the symmetry of Eq (14) follows from the symmetry conditions of the vector field (15):

$$\Xi^{\left(4\right)}V(\zeta ){|}_{\lambda =0}=0,\;\;\;\eta_{c_{1}\ldots c_{k}}^{\left(q\right)}=D_{c_{s}}\eta_{c_{1}\ldots c_{q-1}}^{\left(q-1\right)}-(D_{c_{q}}\xi^{q})u_{c_{1}\ldots c_{q-1}s},\;c_{s}=1,\ldots ,n\text{ for }s=1,\ldots ,q,\;\text{with }\;c_{1},\ldots ,c_{q}=\{\vartheta ,\tau \},\;\;q=2,3,4.\;V(\zeta )=-S{ℭ}_{0}\mu^{\tau \tau }\frac{\partial }{\partial W_{\tau \tau }}+\beta^{2}\mu^{\vartheta \vartheta }\frac{\partial }{\partial W_{\vartheta \vartheta }}+{ℜ}_{2}{ℭ}_{0}\beta^{2}\mu^{\tau \vartheta \vartheta }\frac{\partial }{\partial W_{\tau \vartheta \vartheta }}-{ℜ}_{1}\;{ℭ}_{0}\mu^{\tau }\frac{\partial }{\partial W_{\tau }}+2\alpha {ℜ}_{1}{ℭ}_{0}W\mu^{\tau }\frac{\partial }{\partial W_{\tau }}+2\alpha {ℜ}_{1}{ℭ}_{0}W\mu \frac{\partial W}{\partial \tau },\;\lambda =-SC_{0}\frac{\partial^{2}W}{\partial \tau^{2}}+\beta^{2}\frac{\partial^{2}W}{\partial \vartheta^{2}}+R_{2}C_{0}\frac{\partial }{\partial \tau }(\beta^{2}\frac{\partial^{2}W}{\partial \vartheta^{2}})-R_{1}C_{0}\frac{\partial W}{\partial \tau }+2\alpha R_{1}C_{0}W\frac{\partial W}{\partial \tau }.$$ (16)

$D_{c_{q}}$ (q=1,2,3,4) are complete operators concerning ϑ and *τ*.

**Theorem 1:** Eq (14) possesses a two-dimensional Lie algebra spanned by two generators [31]:

$$\Xi_{1}=\frac{\partial }{\partial \tau },\;\;\Xi_{2}=\frac{\partial }{\partial \vartheta }.$$ (17)

Using Theorem 1 along with the related ODEs and initial conditions:

$$\frac{d\tau^{⋆}}{d\varrho }=\Theta_{b}(\vartheta^{⋆},\tau^{⋆},W^{⋆}),\;\;\;\bar{\tau }{|}_{\varrho =0}=\tau ,\;\frac{d\vartheta^{⋆}}{d\varrho }=\Theta_{a}(\vartheta^{⋆},\tau^{⋆},W^{⋆}),\;\;\;\bar{\vartheta }{|}_{\varrho =0}=\vartheta .$$ (18)

We obtain one-parameter groups $G_{c}$ using $\Xi_{c}$:

$$G_{1}:(\vartheta ,\tau ,W)\to (\vartheta ,\tau +\varrho ,W),\;G_{2}:(\vartheta ,\tau ,W)\to (\vartheta +\varrho ,\tau ,W).$$ (19)

This leads to the following result.

**Theorem 2:** If W(ϑ,τ) is a solution of Eq (14), another solution is given by [32]:

$$H_{1}:W(\vartheta ,\tau )=(\vartheta ,\tau -\varrho ,W),\;H_{2}:W(\vartheta ,\tau )=(\vartheta -\varrho ,\tau ,W).\;$$ (20)

Using the Lie bracket definition $[\Xi_{\vartheta },\Xi_{\tau }]=\Xi_{\vartheta }\Xi_{\tau }-\Xi_{\tau }\Xi_{\vartheta }$, the commutators are given by:

$$[\Xi_{i},\Xi_{j}]=[\Xi_{j},\Xi_{i}]=0,\;\;i,j=1,2.$$ (21)

**Theorem 3:** A two-dimensional Lie symmetry algebra G is generated by $\Xi_{c}$ (c=1,2) in Theorem 1. Next, we determine the adjoint representation of the vector fields using the Lie series [32]:

$$\text{Adj}(e^{\varrho \Xi_{i}})\Xi_{j}=\Xi_{j}-\varrho [\Xi_{i},\Xi_{j}]+\frac{\varrho^{2}}{2}[\Xi_{i},[\Xi_{i},\Xi_{j}]]-\cdots ,\;\varrho \in \mathbb{R}.$$ (22)

Applying system (21), we obtain:

$$Adj(e^{\varrho \Xi_{i}})\Xi_{i}=\Xi_{i},\;i=1,2.\;Adj(e^{\varrho \Xi_{i}})\Xi_{j}=\Xi_{j},\;i\neq j=1,2.$$ (23)

**Theorem 4:** A one-parameter optimal system [33] for the Lie algebra G is formed by the operators $\left\{\Xi_{1},\Xi_{2},\Xi_{1}+\Psi \Xi_{2}\right\}$ with an arbitrary constant Ψ.

## 3 Exact solutions via symmetry reductions

This section focuses on analyzing symmetry reductions and exact solutions for Eq (14).

### 3.1 The operator $\Xi_{1}$

Utilizing the generator $\Xi_{1}=\frac{\partial }{\partial \vartheta }$, one derives

W(ϑ,τ)=𝒥(φ). (24)

Setting φ=τ and inserting Eq (24) into Eq (14), we obtain the ODE:

$$2C_{0}𝒥{𝒥}^{\prime }R_{1}\alpha -C_{0}S{𝒥}^{\prime \prime }-C{𝒥}^{\prime }R_{1}=0.$$ (25)

We solve Eq (25) in maple and obtain the following solution:

$$𝒥(\varphi )=\frac{2\tan(\frac{\sqrt{R_{1}S\alpha f_{1}}(f_{2}+\varphi )}{S})\sqrt{R_{1}S\alpha C_{0}}+R_{1}}{2\alpha R_{1}}.$$ (26)

Substituting Eq (26) into Eq (24) yields the solution of Eq (14):

$$W(\vartheta ,\;\tau )=\frac{2\tan(\frac{\sqrt{R_{1}S\alpha f_{1}}(f_{2}+\tau )}{S})\sqrt{R_{1}S\alpha C_{0}}+R_{1}}{2\alpha R_{1}}.$$ (27)

### 3.2 The operator $\Xi_{2}$

Based on the generator $\Xi_{2}=\frac{\partial }{\partial \tau }$, we have :

W(ϑ,τ)=𝒥(φ). (28)

With φ=ϑ, substituting Eq (28) into Eq (14) yields the following ODE:

$$\beta^{2}{𝒥}^{\prime \prime }=0.$$ (29)

The obtained solution for Eq (29) is:

$$𝒥(\varphi )={ℬ}_{1}\varphi +{ℬ}_{2}.$$ (30)

Substituting Eq (30) into Eq (28) yields the solution of Eq (14):

$$W(\vartheta ,\;\tau )={ℬ}_{1}\vartheta +{ℬ}_{2}.$$ (31)

### 3.3 The operator $a_{1}\Xi_{1}+a_{2}\Xi_{2}$

From $a_{1}\Xi_{1}+a_{2}\Xi_{2}=a_{1}\frac{\partial }{\partial \vartheta }+a_{2}\frac{\partial }{\partial \tau }$, it follows that:

W(ϑ,τ)=𝒥(φ). (32)

Taking $\varphi =a_{2}(\vartheta -\chi \tau )$, substituting Eq (32) into Eq (14) leads to the ODE:

$$-SC_{0}{𝒥}^{\prime \prime }\chi^{2}a_{2}^{2}+\beta^{2}a_{2}^{2}{𝒥}^{\prime \prime }-R_{2}C_{0}\beta^{2}\chi a_{2}^{3}{𝒥}^{\prime \prime \prime }+R_{1}C_{0}a_{2}\chi {𝒥}^{\prime }-2\alpha R_{1}C_{0}\chi a_{2}{𝒥}^{\prime }𝒥=0.$$ (33)

Integrating Eq (33) with respect to φ, we obtain the following ODE:

$${𝒥}^{\prime }(\beta^{2}a_{2}^{2}-SC_{0}\chi^{2}a_{2}^{2})-R_{2}C_{0}\beta^{2}\chi a_{2}^{3}{𝒥}^{\prime \prime }+R_{1}C_{0}a_{2}\chi 𝒥-\alpha R_{1}C_{0}\chi a_{2}{𝒥}^{2}=0.$$ (34)

### 3.4 Exploring wave solution behaviors

In this part, We aim to derive solitary wave solutions for Eq (14) using the tanh method. A detailed explanation of the method is available in [34]. The balancing number (P) between the higher-order derivative and the nonlinear terms ${𝒥}^{\prime \prime }$ and ${𝒥}^{2}$ to determine the value from Eq (34). We apply the balancing procedure to the ODE in Eq (34) and obtain P=2. Consequently, from $𝒥(\varphi )=\sum_{y=0}^{P}{ℰ}_{y}{𝒦}^{y}(\varphi )$, we get:

$$𝒥(\varphi )={ℰ}_{0}+{ℰ}_{1}𝒦(\varphi )+{ℰ}_{2}{𝒦}^{2}(\varphi ),\;\;\;{ℰ}_{2}\;\neq \;0.$$ (35)

By inserting Eq (35) into Eq (34), we derive a system of algebraic equations. Solving these equations using Maple, we obtain:

$$\left\{ \begin{array}{c} {ℰ}_{0}=\frac{3}{2\alpha },\;{ℰ}_{1}=0,\;{ℰ}_{2}=\frac{-3}{2\alpha },\;\Psi =\frac{1}{\sqrt{{ℭ}_{0}S}},\;a_{2}=\frac{\sqrt{{ℜ}_{1}}}{2\sqrt{\Psi }\beta },\;\;\\ W(\vartheta ,\tau )=\frac{3}{2\alpha }+\frac{\;-3}{2\alpha }\;(\tanh[a_{2}(\vartheta -\chi \tau )]{)}^{2}. \end{array}\right.$$ (36)

### 3.5 Graphical representation

**Fig 4:**
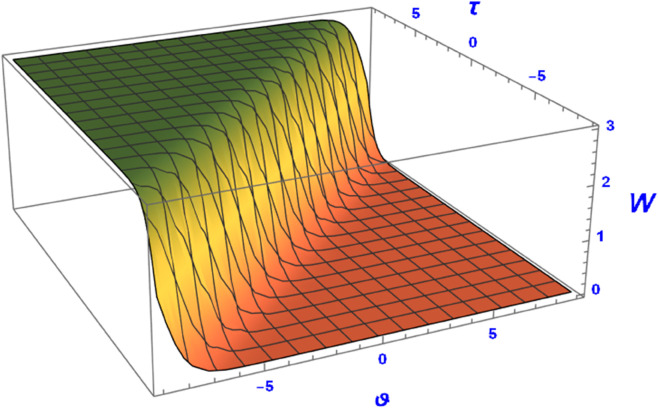
Anti-kink soliton profile of W(ϑ,τ) from Eq (36) for positive wave speed using 3D plot.

**Fig 5:**
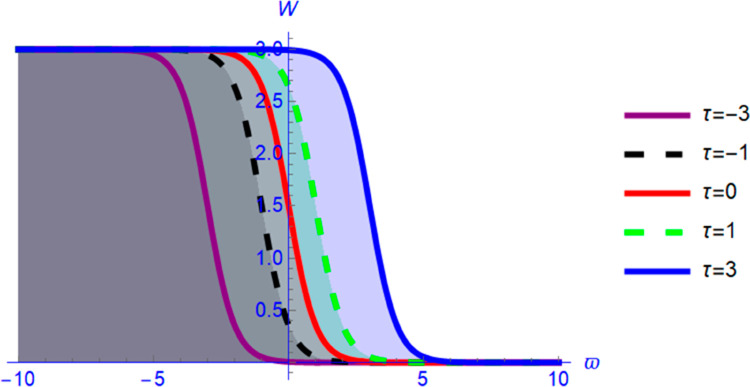
Anti-kink soliton profile of W(ϑ,τ) from Eq (36) for positive wave speed using 2D plot.

**Fig 6:**
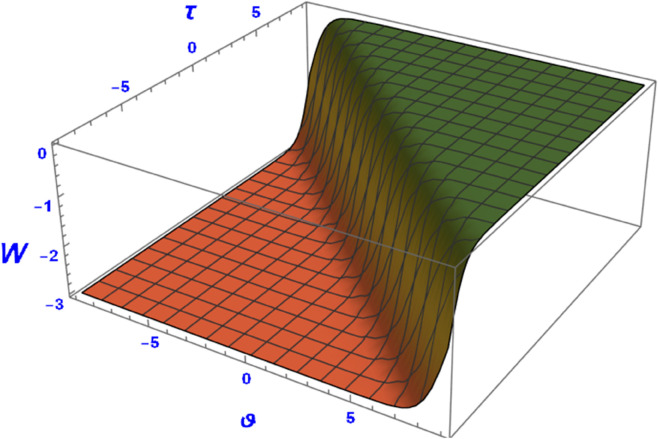
Kink soliton profile of W(ϑ,τ) from Eq (36) for negative wave speed.

**Fig 7:**
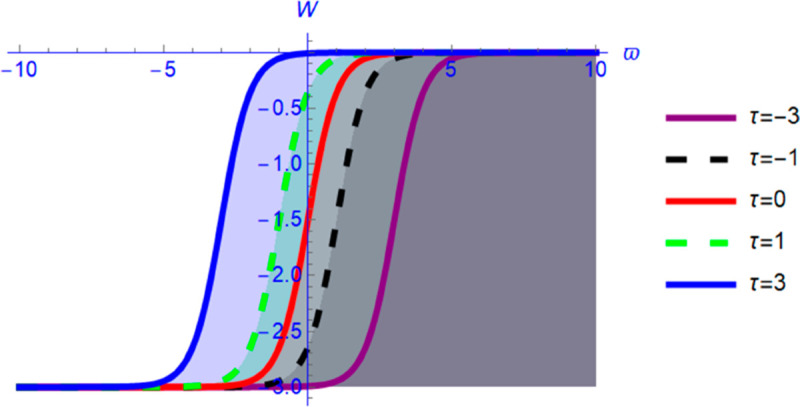
Kink soliton profile of W(ϑ,τ) from Eq (36) for negative wave speed.

This section presents graphical representations of the obtained solutions. By selecting appropriate parameter values, we derive both 2D and 3D visualizations of Eq (36) with *a*_2_ = 0.56 and α=1.56. As shown in Figs 4 and 5, the solution exhibits a kink profile for χ=0.63, while for χ=−0.63, the corresponding representations in Figs 6 and 7 reveal an anti-kink-shaped solution. Kink and anti-kink solitons play a vital role in nonlinear wave dynamics due to their stability and energy-preserving nature. They model signal transmission in optical fibers, energy transport in plasmas, and biological processes like DNA dynamics. In condensed matter physics, they describe domain walls in ferromagnets and phase transitions in superconductors. Additionally, they appear in fluid dynamics and cosmology, representing shock waves and topological defects. Their robustness makes them essential in various scientific and engineering applications [35–38].

## 4 Bifurcation analysis of Eq (14)

Bifurcation occurs in dynamical systems when slight changes in system parameters or conditions lead to a qualitative shift in behavior. As parameters surpass critical thresholds, known as bifurcation points, the system undergoes transformations such as the emergence or disappearance of stable fixed points, limit cycles, or chaotic dynamics. These transitions may cause the system to shift between stability and instability or give rise to new dynamic patterns. Eq (34) written as a dynamical system is [39]:

$$\left\{ \begin{array}{c} \frac{d𝒥}{d\varphi }=𝒱\\ \frac{d𝒱}{d\varphi }={𝔉}_{1}𝒱+{𝔉}_{2}𝒥-{𝔉}_{3}{𝒥}^{2},\\ {𝔉}_{1}=\frac{\beta^{2}-SC_{0}\chi^{2}}{R_{2}C_{0}\beta^{2}\chi a_{2}},\;\;{𝔉}_{2}=\frac{R_{1}}{R_{2}\beta^{2}a_{2}^{2}},\;\;{𝔉}_{3}=\frac{\alpha R_{1}}{R_{2}\beta^{2}a_{2}^{2}}\cdot \end{array}\right.$$ (37)

System (37) has the following fixed points along the 𝒥−axis :

$${ℬ}_{1}=(0,0),\;\;{ℬ}_{2}=(\frac{{𝔉}_{2}}{{𝔉}_{3}},0).$$ (38)

The Jacobian matrix for system (37) is obtained through the following computations [40]:

$$\gamma (𝒥,𝒱)=[\begin{array}{cc} 0 & 1\\ {𝔉}_{2}-2{𝔉}_{3}𝒥 & {𝔉}_{1} \end{array}]$$ (39)

$$\gamma (𝒥,𝒱)=2{𝔉}_{3}𝒥-{𝔉}_{2}.$$ (40)

**Remark:** The point (𝒥,𝒱) is a saddle if γ(𝒥,𝒱)<0, a center if γ(𝒥,𝒱)>0, and a cusp if γ(𝒥,𝒱)=0. The nature of system (37) at critical points is determined accordingly [41]. The detailed results of the global phase portraits are discussed in Tables 1–2 and illustrated in Figs 8–19.

**Fig 8:**
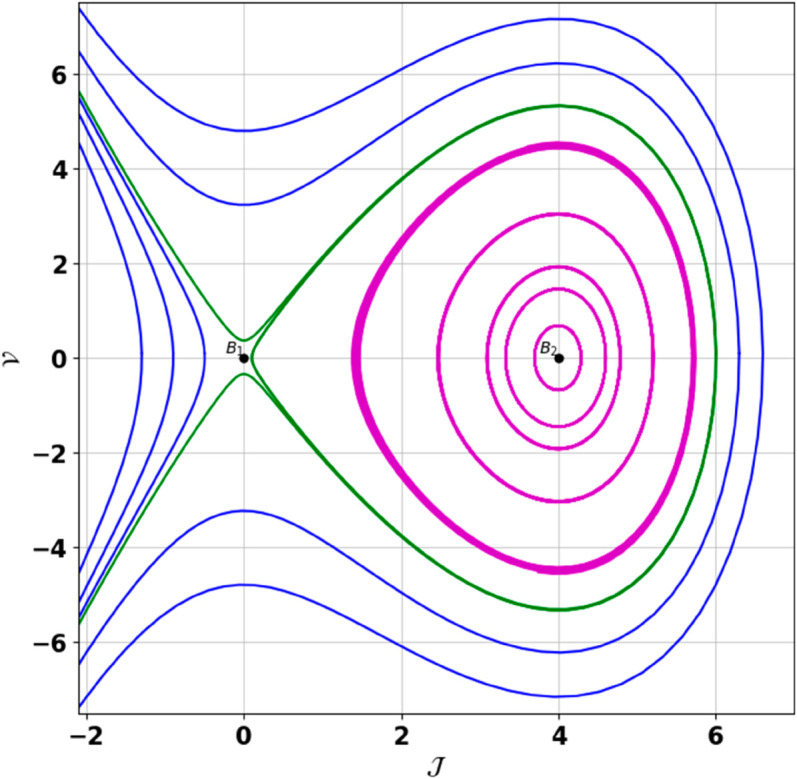
Global phase portraits of the dynamical system (37) for positive ${𝔉}_{2}$ and ${𝔉}_{3}$, when ${𝔉}_{1}$ is absent.

**Fig 9:**
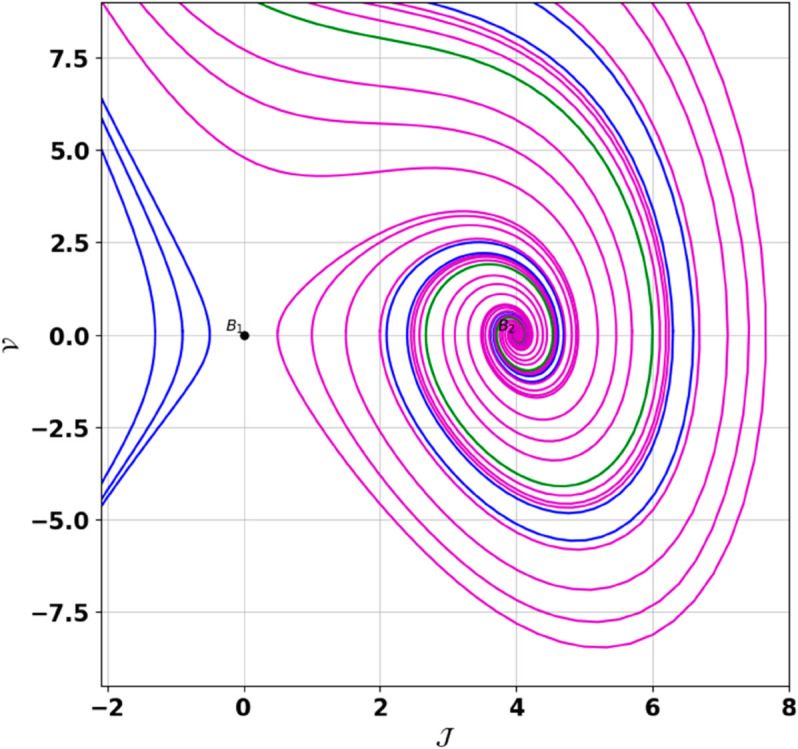
Global phase portraits of the dynamical system (37) for positive ${𝔉}_{2}$ and ${𝔉}_{3}$, when ${𝔉}_{1}$ is positive.

**Fig 10:**
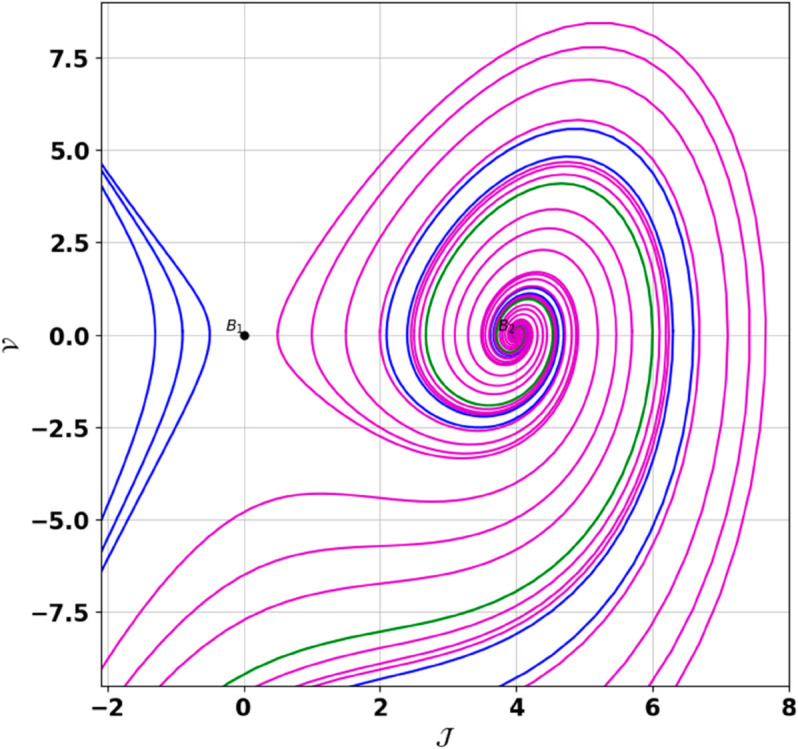
Global phase portraits of the dynamical system (37) for positive ${𝔉}_{2}$ and ${𝔉}_{3}$, when ${𝔉}_{1}$ is negative.

**Fig 11:**
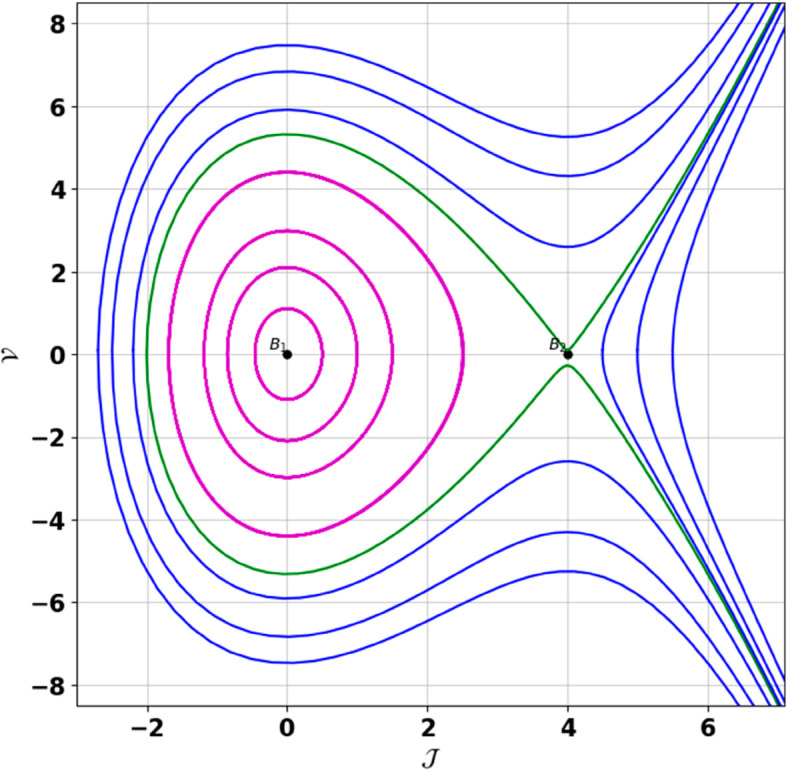
Global phase portraits of the dynamical system (37) for negative ${𝔉}_{2}$ and ${𝔉}_{3}$, when ${𝔉}_{1}$ is absent.

**Fig 12:**
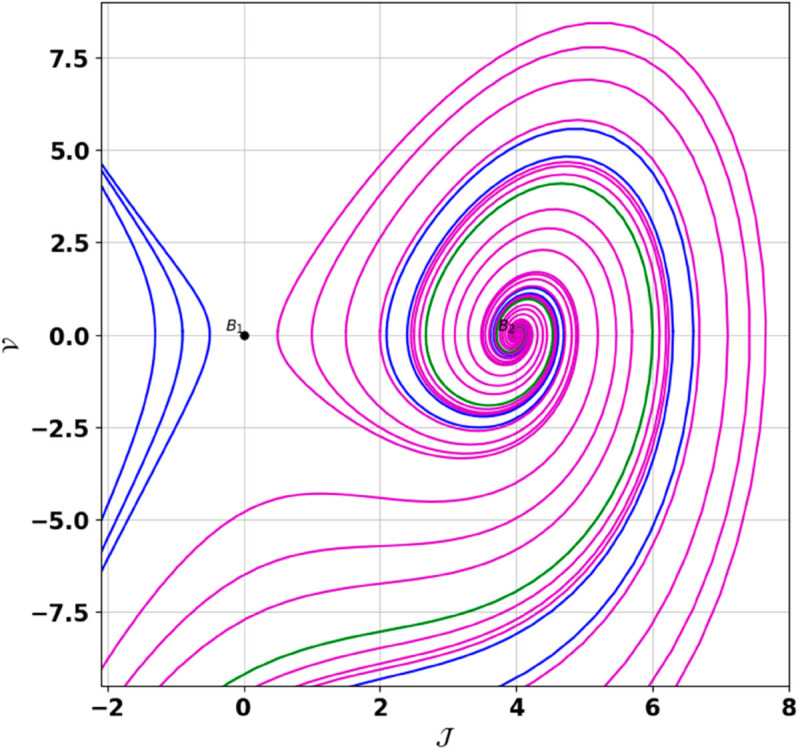
Global phase portraits of the dynamical system (37) for negative ${𝔉}_{2}$ and ${𝔉}_{3}$, when ${𝔉}_{1}$ is positive.

**Fig 13:**
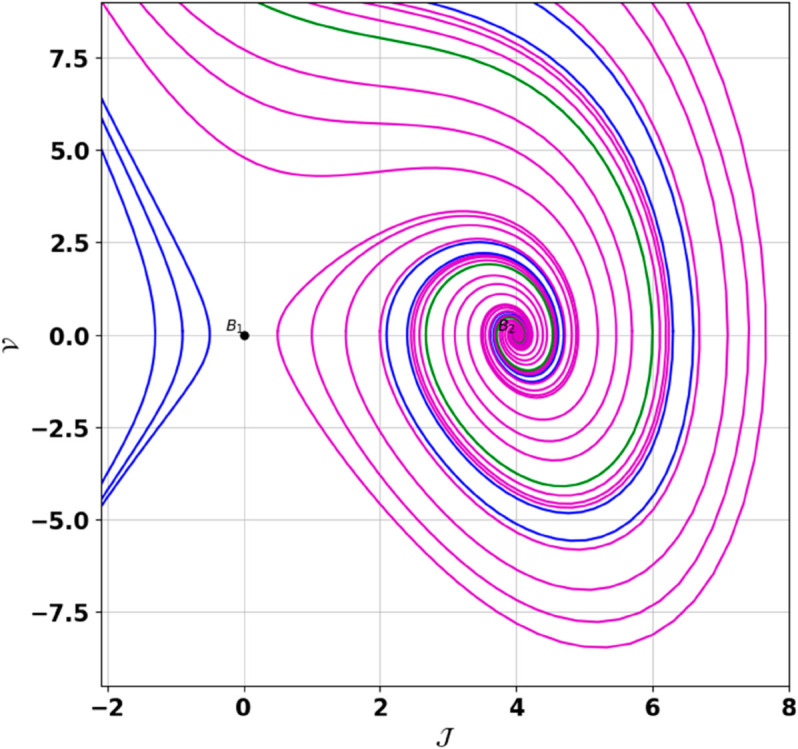
Global phase portraits of the dynamical system (37) for negative ${𝔉}_{2}$ and ${𝔉}_{3}$, when ${𝔉}_{1}$ is negative.

**Fig 14:**
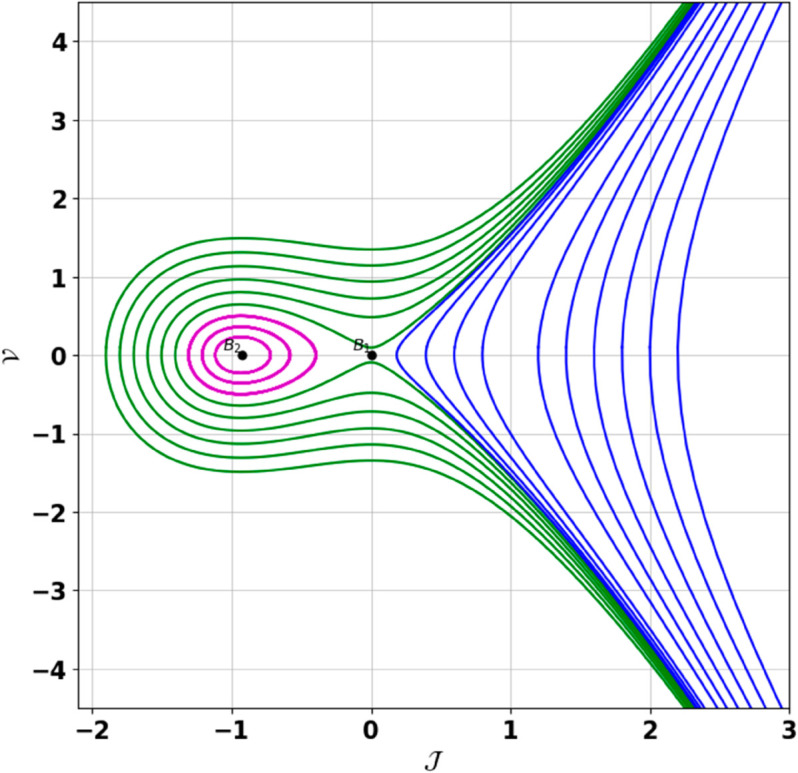
Global phase portraits of the dynamical system (37) for ${𝔉}_{2}>0$ and ${𝔉}_{3}<0$, when ${𝔉}_{1}$ is absent.

**Fig 15:**
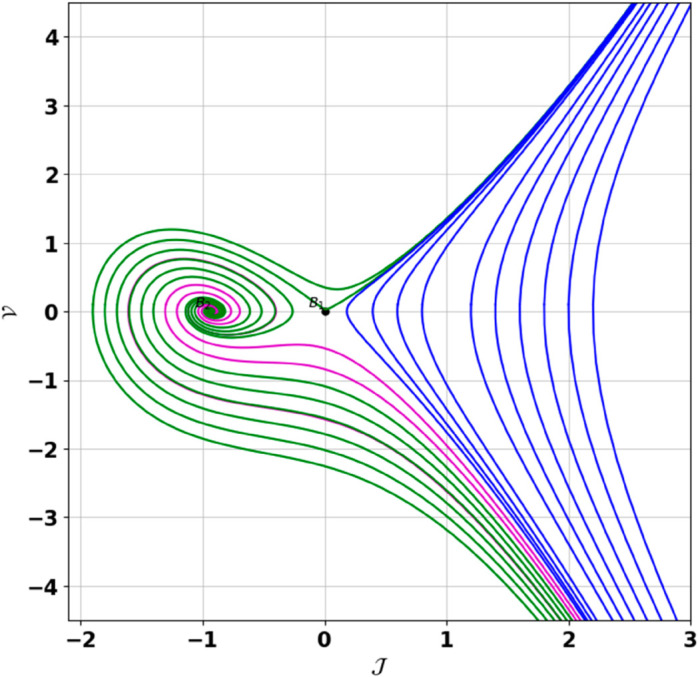
Global phase portraits of the dynamical system (37) for ${𝔉}_{2}>0$ and ${𝔉}_{3}<0$, when ${𝔉}_{1}$ is positive.

**Fig 16:**
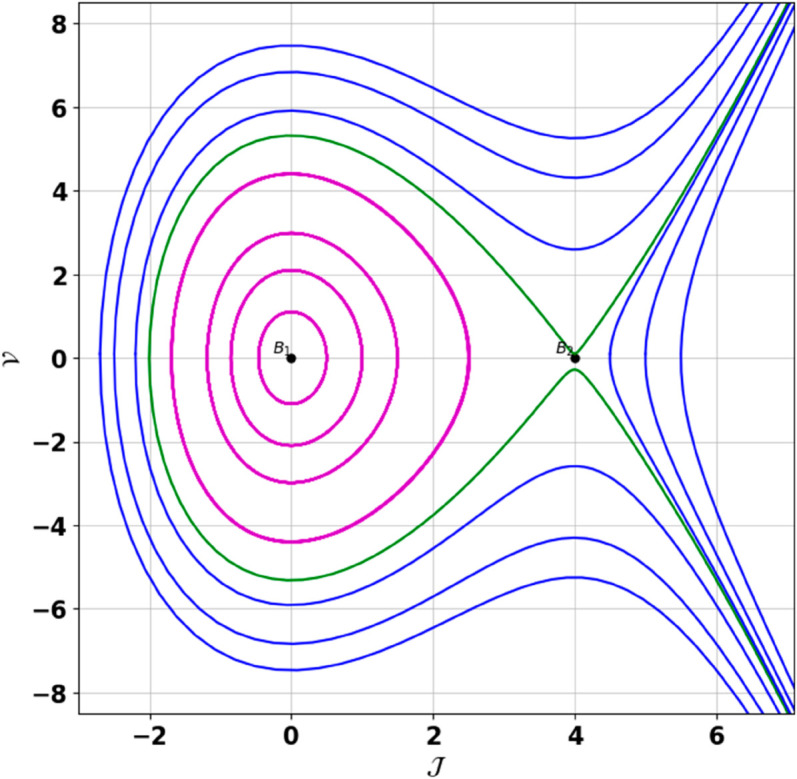
Global phase portraits of the dynamical system (37) for ${𝔉}_{2}>0$ and ${𝔉}_{3}<0$, when ${𝔉}_{1}$ is negative.

**Fig 17:**
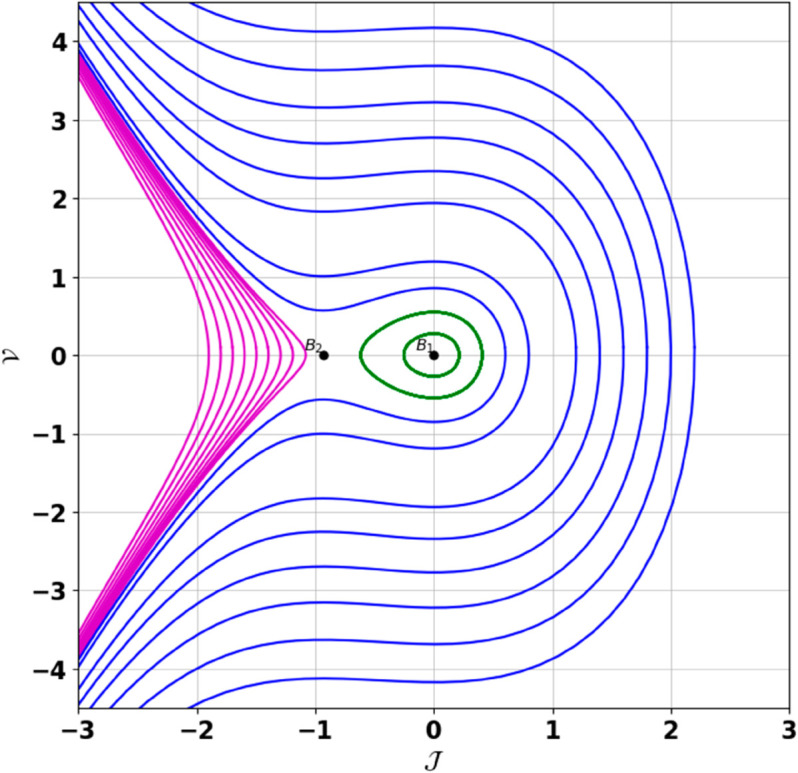
Global phase portraits of the dynamical system (37) for ${𝔉}_{2}<0$ and ${𝔉}_{3}>0$, when ${𝔉}_{1}$ is absent.

**Fig 18:**
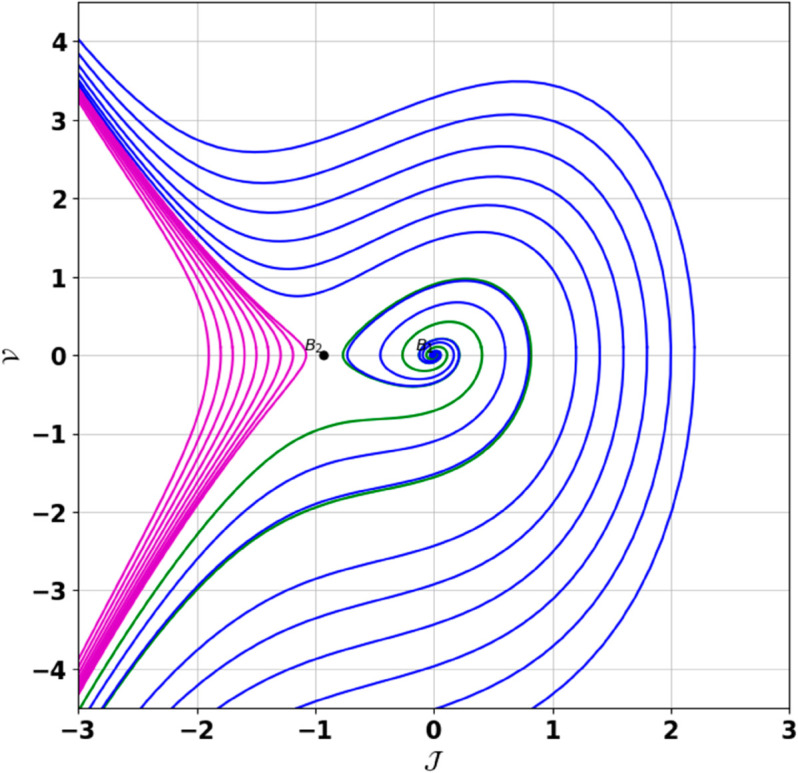
Global phase portraits of the dynamical system (37) for ${𝔉}_{2}<0$ and ${𝔉}_{3}>0$, when ${𝔉}_{1}$ is positive.

**Fig 19:**
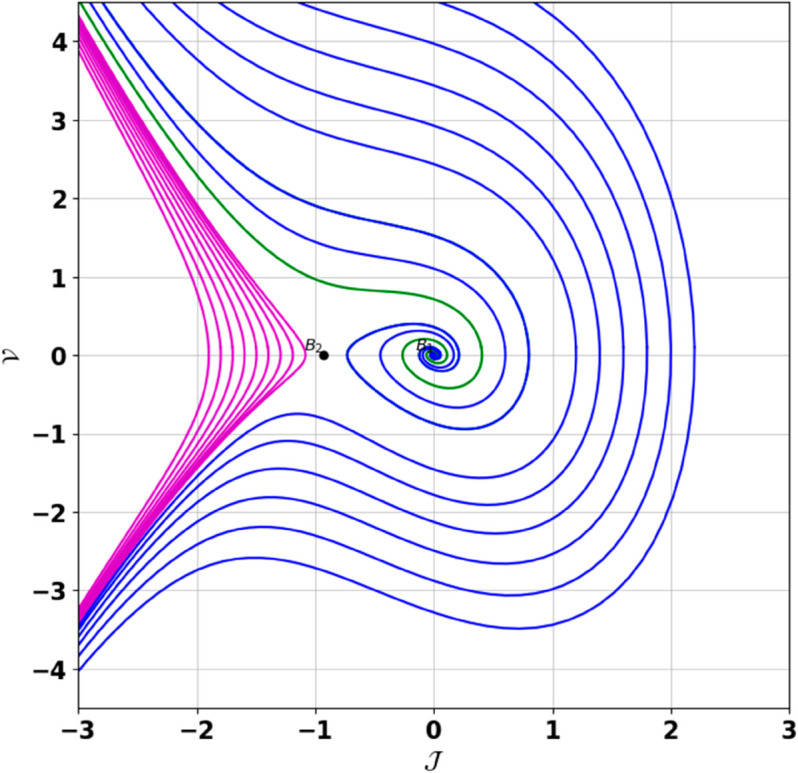
Global phase portraits of the dynamical system (37) for ${𝔉}_{2}<0$ and ${𝔉}_{3}>0$, when ${𝔉}_{1}$ is negative.

**Table 1:** Conditions and parameter values corresponding to Global Phase Portraits.

Figure	Conditions	Values
8	${𝔉}_{2}>0,\;{𝔉}_{3}>0,\;{𝔉}_{1}=0$	${𝔉}_{2}=5.287,\;{𝔉}_{3}=1.3219,\;{𝔉}_{1}=0$
9	${𝔉}_{2}>0,\;{𝔉}_{3}>0,\;{𝔉}_{1}>0$	${𝔉}_{2}=5.287,\;{𝔉}_{3}=1.3219,\;{𝔉}_{1}=0.75$
10	${𝔉}_{2}>0,\;{𝔉}_{3}>0,\;{𝔉}_{1}<0$	${𝔉}_{2}=5.287,\;{𝔉}_{3}=1.3219,\;{𝔉}_{1}=-0.75$
11	${𝔉}_{2}<0,\;{𝔉}_{3}<0,\;{𝔉}_{1}=0$	${𝔉}_{2}=-5.287,\;{𝔉}_{3}=-1.3219,\;{𝔉}_{1}=0$
12	${𝔉}_{2}<0,\;{𝔉}_{3}<0,\;{𝔉}_{1}>0$	${𝔉}_{2}=-5.287,\;{𝔉}_{3}=-1.3219,\;{𝔉}_{1}=0.35$
13	${𝔉}_{2}<0,\;{𝔉}_{3}<0,\;{𝔉}_{1}<0$	${𝔉}_{2}=-5.287,\;{𝔉}_{3}=-1.3219,\;{𝔉}_{1}=-0.35$
14	${𝔉}_{2}>0,\;{𝔉}_{3}<0,\;{𝔉}_{1}=0$	${𝔉}_{2}=5.287,\;{𝔉}_{3}=-1.3219,\;{𝔉}_{1}=0$
15	${𝔉}_{2}>0,\;{𝔉}_{3}<0,\;{𝔉}_{1}>0$	${𝔉}_{2}=5.287,\;{𝔉}_{3}=-1.3219,\;{𝔉}_{1}=0.55$
16	${𝔉}_{2}>0,\;{𝔉}_{3}<0,\;{𝔉}_{1}<0$	${𝔉}_{2}=5.287,\;{𝔉}_{3}=-1.3219,\;{𝔉}_{1}=-0.55$
17	${𝔉}_{2}<0,\;{𝔉}_{3}>0,\;{𝔉}_{1}=0$	${𝔉}_{2}=-5.287,\;{𝔉}_{3}=1.3219,\;{𝔉}_{1}=0$
18	${𝔉}_{2}<0,\;{𝔉}_{3}>0,\;{𝔉}_{1}>0$	${𝔉}_{2}=-5.287,\;{𝔉}_{3}=1.3219,\;{𝔉}_{1}=0.25$
19	${𝔉}_{2}<0,\;{𝔉}_{3}>0,\;{𝔉}_{1}<0$	${𝔉}_{2}=-5.287,\;{𝔉}_{3}=1.3219,\;{𝔉}_{1}=-0.25$

**Table 2:** Equilibrium points behaviors, and trajectory types of global phase portraits.

Figure	Equilibrium Points	Behavior	Trajectory Type
8	${ℬ}_{1}=(0,0),\;{ℬ}_{2}=(4,0)$	${ℬ}_{1}$: Saddle, ${ℬ}_{2}$: Center	Pink: closed periodic orbit, Green: homoclinic orbit
9	${ℬ}_{1}=(0,0),\;{ℬ}_{2}=(4,0)$	${ℬ}_{1}$: Saddle, ${ℬ}_{2}$: Center	Pink: closed periodic orbit, Green: homoclinic orbit
10	${ℬ}_{1}=(0,0),\;{ℬ}_{2}=(4,0)$	${ℬ}_{1}$: Saddle, ${ℬ}_{2}$: Center	Pink: closed periodic orbit, Green: homoclinic orbit
11	${ℬ}_{1}=(0,0),\;{ℬ}_{2}=(4,0)$	${ℬ}_{1}$: Center, ${ℬ}_{2}$: Saddle	Pink: closed periodic orbit, Green: homoclinic orbit
12	${ℬ}_{1}=(0,0),\;{ℬ}_{2}=(4,0)$	${ℬ}_{1}$: Center, ${ℬ}_{2}$: Saddle	Pink: closed periodic orbit, Green: homoclinic orbit
13	${ℬ}_{1}=(0,0),\;{ℬ}_{2}=(4,0)$	${ℬ}_{1}$: Center, ${ℬ}_{2}$: Saddle	Pink: closed periodic orbit, Green: homoclinic orbit
14	${ℬ}_{1}=(0,0),\;{ℬ}_{2}=(-0.93,0)$	${ℬ}_{1}$: Saddle, ${ℬ}_{2}$: Center	Pink: closed periodic orbit, Green: homoclinic orbit
15	${ℬ}_{1}=(0,0),\;{ℬ}_{2}=(-0.93,0)$	${ℬ}_{1}$: Saddle, ${ℬ}_{2}$: Center	Pink: closed periodic orbit, Green: homoclinic orbit
16	${ℬ}_{1}=(0,0),\;{ℬ}_{2}=(-0.93,0)$	${ℬ}_{1}$: Saddle, ${ℬ}_{2}$: Center	Pink: closed periodic orbit, Green: homoclinic orbit
17	${ℬ}_{1}=(0,0),\;{ℬ}_{2}=(-0.93,0)$	${ℬ}_{1}$: Center, ${ℬ}_{2}$: Saddle	Green: closed periodic orbit, Blue: homoclinic orbit
18	${ℬ}_{1}=(0,0),\;{ℬ}_{2}=(-0.93,0)$	${ℬ}_{1}$: Center, ${ℬ}_{2}$: Saddle	Green: closed periodic orbit, Blue: homoclinic orbit
19	${ℬ}_{1}=(0,0),\;{ℬ}_{2}=(-0.93,0)$	${ℬ}_{1}$: Center, ${ℬ}_{2}$: Saddle	Green: closed periodic orbit, Blue: homoclinic orbit

## 5 Hamilton analysis of Eq (14)

Using Hamilton’s equations from classical mechanics, the system’s behavior can be effectively analyzed and understood [42]

$$\frac{dA_{1}}{d\tau }={\mathbb{P}}_{1}(𝒥,𝒱),\;\;\frac{d{ℬ}_{1}}{d\tau }={𝕐}_{1}(𝒥,𝒱).$$ (41)

A system is called Hamiltonian if a function A(𝒥,𝒱) exists that satisfies:

$${\mathbb{P}}_{1}=\frac{\partial A}{\partial 𝒱}\;\mathrm{and}\;{𝕐}_{1}=-\frac{\partial A}{\partial 𝒥}.$$ (42)

The Hamiltonian function of the system is denoted by A [43].

**Definition:** For the dynamical system (41) to be classified as Hamiltonian, specific conditions must be met:

$$\frac{\partial {\mathbb{P}}_{1}}{\partial 𝒥}+\frac{\partial {𝕐}_{1}}{\partial 𝒱}=0.$$ (43)

Eq (37) qualifies as a Hamiltonian dynamical system since it meets the required conditions for the state equations:


$$\frac{\partial }{\partial 𝒥}(\frac{d𝒥}{d\varphi })+\frac{\partial }{\partial 𝒱}(\frac{d𝒱}{d\varphi })=0.$$

The Hamiltonian corresponding to system (37) is given by [44]:

$$A(𝒥,𝒱)=\frac{{𝔉}_{1}{𝒱}^{2}}{2}-\frac{{𝔉}_{2}{𝒥}^{2}}{2}-\frac{{𝔉}_{3}{𝒥}^{3}}{3}=z_{1},$$ (44)

where *z*_1_ is a real parameter.

## 6 Investigation of chaotic behavior

Understanding the intrinsic dynamics of Eq (34) is crucial. This section presents a detailed examination of Eq (37)’s response to noise-induced perturbations and its display of chaotic behavior. The analysis follows the proposed model [45]:

$$\left\{ \begin{array}{c} \frac{d𝒥}{d\varphi }=𝒱\\ \frac{d𝒱}{d\varphi }={𝔉}_{2}𝒥-{𝔉}_{3}{𝒥}^{2}+\Delta \cos(\kappa \varphi ),\\ {𝔉}_{2}=\frac{R_{1}}{R_{2}\beta^{2}a_{2}^{2}},\;\;{𝔉}_{3}=\frac{\alpha R_{1}}{R_{2}\beta^{2}a_{2}^{2}}\cdot \end{array}\right.$$ (45)

**Fig 20:**
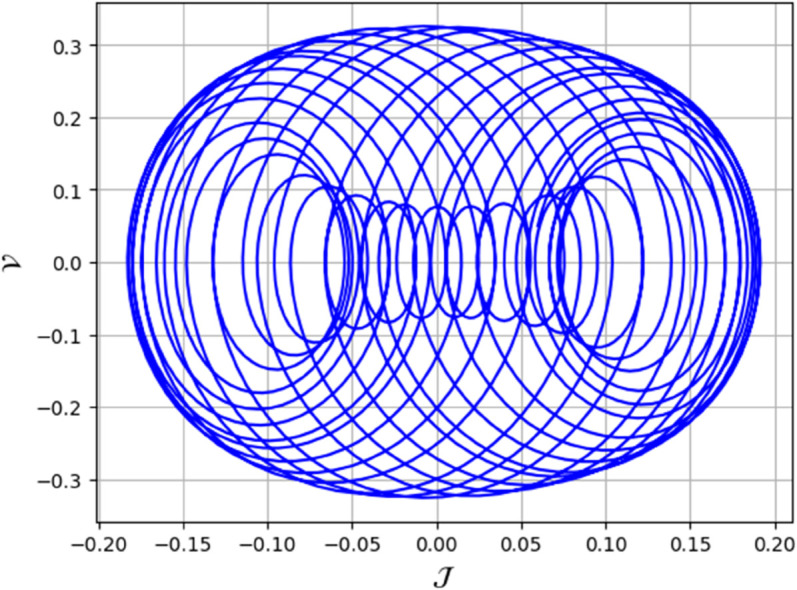
Identification of quasi-periodic behaviour through 2D phase portrait analysis for dynamical system (45) at ${𝔉}_{1}$ = 0.85, ${𝔉}_{2}$ = -0.45, Δ=0.11, and κ=2.28.

**Fig 21:**
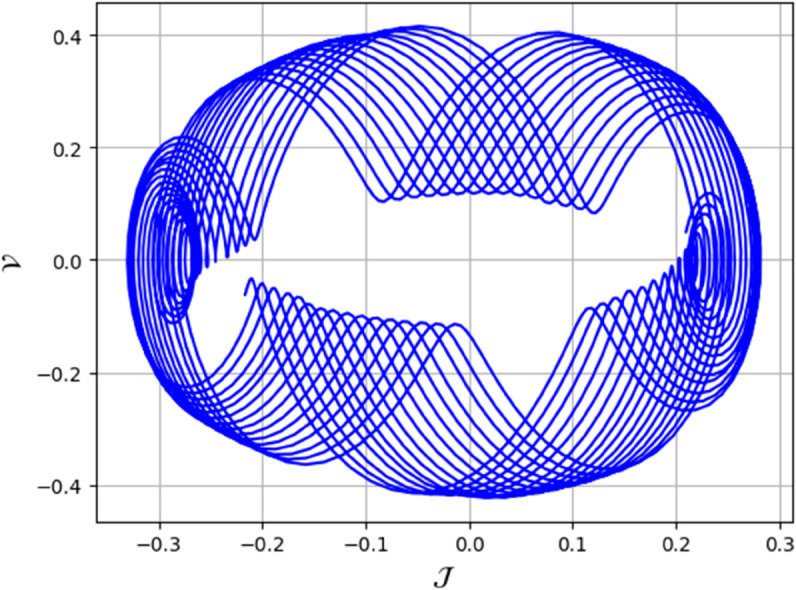
Identification of quasi-periodic behaviour through 2D phase portrait analysis for dynamical system (45) at ${𝔉}_{1}$ = 0.85, ${𝔉}_{2}=-0.45$, Δ=0.33, and κ=2.28.

**Fig 22:**
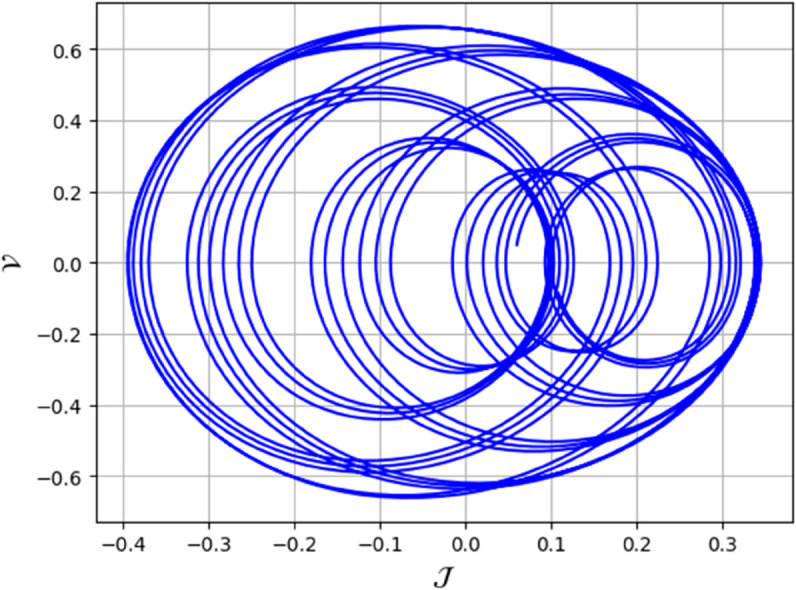
Identification of chaotic behaviour through 2D phase portrait analysis for dynamical system (45) at ${𝔉}_{1}=0.85$, ${𝔉}_{2}$ = -0.45, Δ=0.44, and κ=2.28.

**Fig 23:**
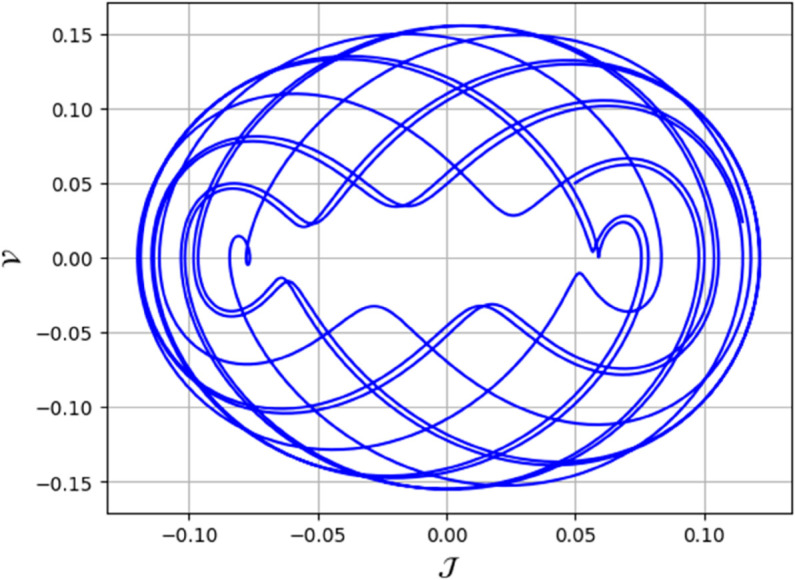
Identification of chaotic behaviour through 2D phase portrait analysis for dynamical system (45) at ${𝔉}_{1}$ = 0.85, ${𝔉}_{2}$ = -0.45, Δ=0.55, and κ=2.28.

**Fig 24:**
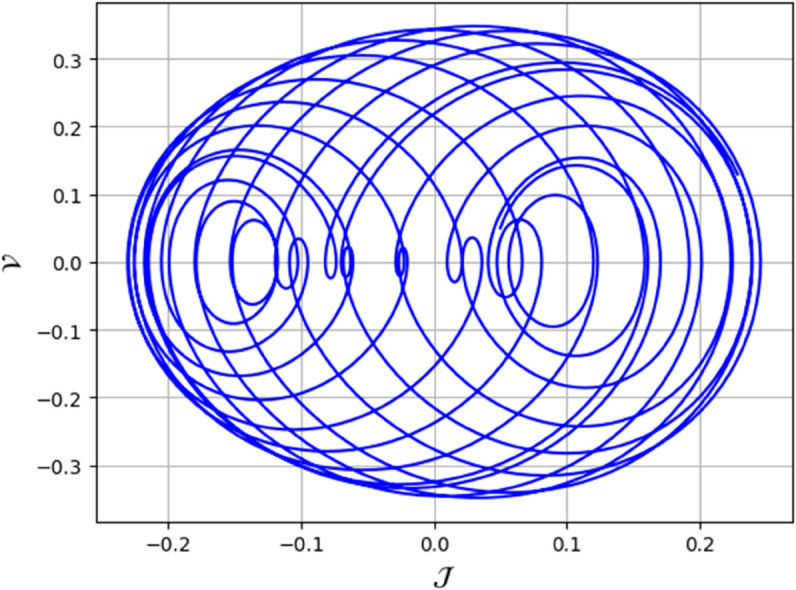
Identification of chaotic behaviour through 2D phase portrait analysis for dynamical system (45) at ${𝔉}_{1}$ = 0.85, ${𝔉}_{2}$ = -0.45, Δ=0.66, and κ=2.28.

**Fig 25:**
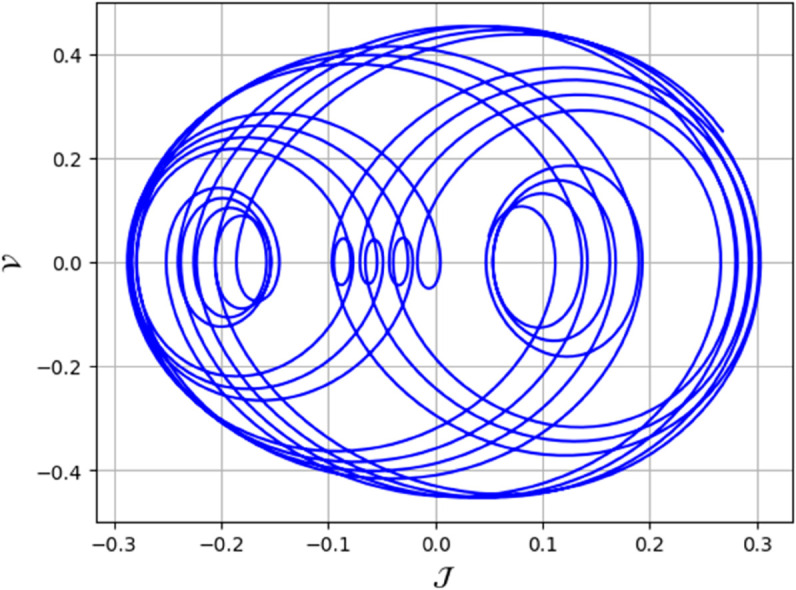
Identification of quasi-periodic and chaotic behaviour through 2D phase portrait analysis for dynamical system (45) at ${𝔉}_{1}$ = 0.85, ${𝔉}_{2}$ = -0.45, Δ=0.77, and κ=2.28.

**Fig 26:**
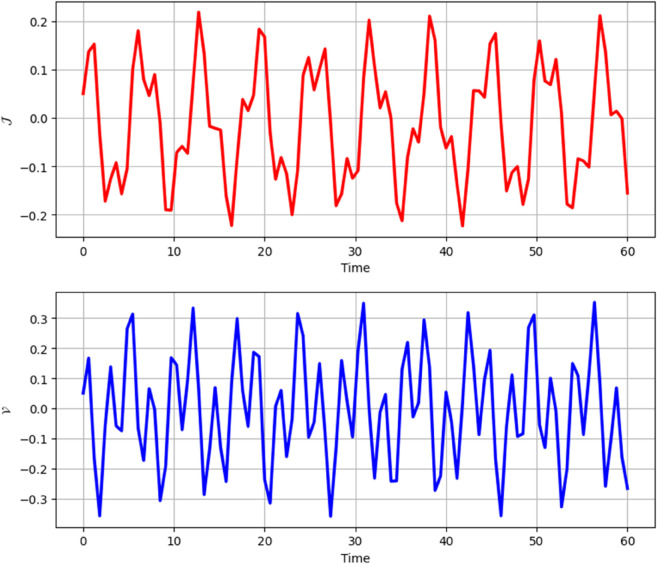
Chaotic behaviour in time analysis of system (45) for Δ=0.33 and κ=2.28 with ${𝔉}_{2}$ > 0, ${𝔉}_{3}$ > 0.

**Fig 27:**
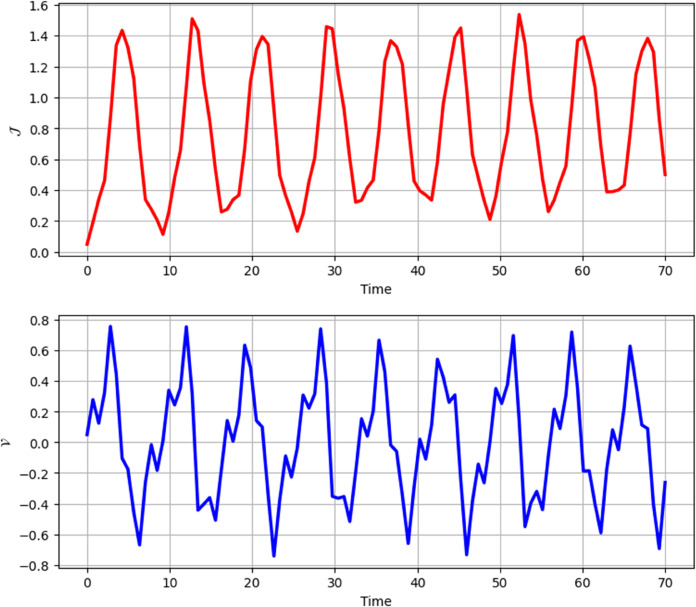
Chaotic behaviour in time analysis of system (45) for Δ=0.33 and κ=2.28 with ${𝔉}_{2}$ < 0, ${𝔉}_{3}$ < 0.

**Fig 28:**
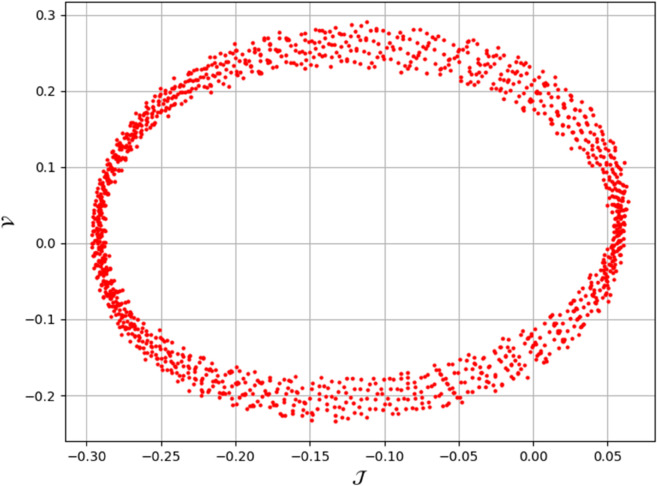
Identification of chaotic behaviour through poincaré map for dynamical system (45) at ${𝔉}_{1}$ = 0.85, Δ=0.11 , ${𝔉}_{2}$ = -0.45, and κ=2.28.

**Fig 29:**
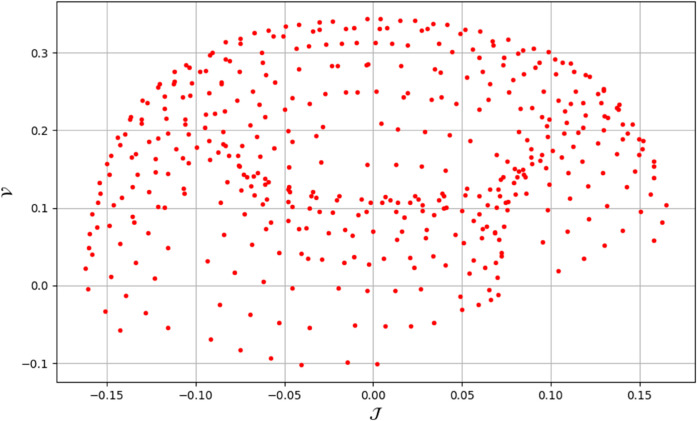
Identification of chaotic behaviour through poincaré map for dynamical system (45) at ${𝔉}_{1}=0.85$, Δ=0.33 , ${𝔉}_{2}=-0.45$, and κ=2.28.

**Fig 30:**
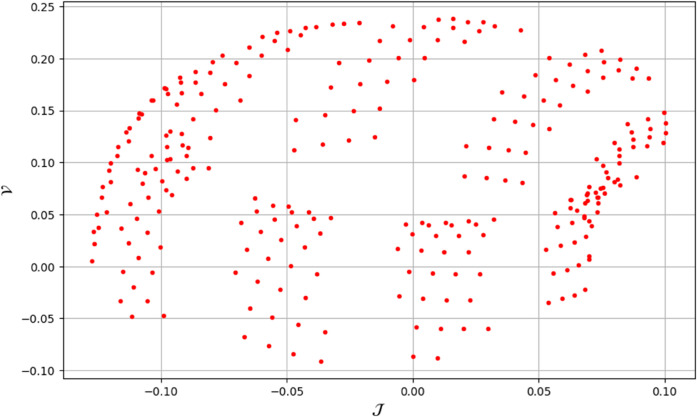
Identification of chaotic behaviour through poincaré map for dynamical system (45) at ${𝔉}_{1}=0.85$, Δ=0.44 , ${𝔉}_{2}=-0.45$, and κ=2.28.

**Fig 31:**
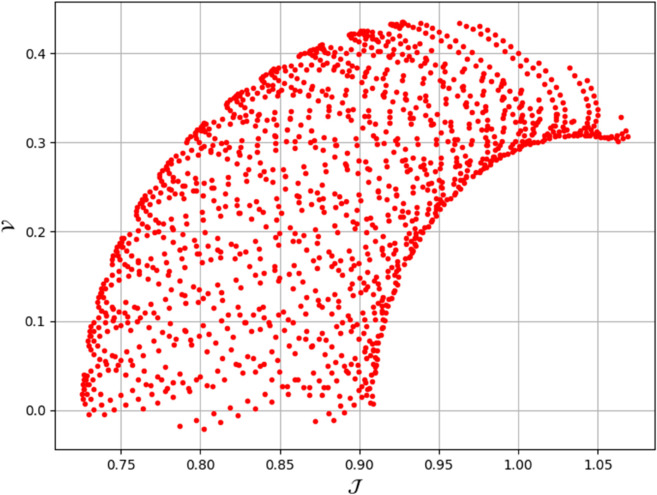
Identification of chaotic behaviour through poincaré map for dynamical system (45) at ${𝔉}_{1}=0.85$, Δ=0.77, ${𝔉}_{2}=-0.45$, and κ=2.28.

**Fig 32:**
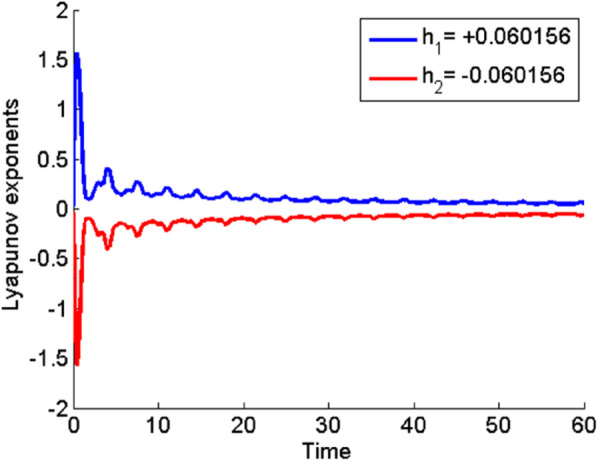
Detection of chaotic behavior through the Lyapunov exponent for dynamical system (45) with ${𝔉}_{1}=0.85$, ${𝔉}_{2}=-0.45$, and κ=2.28.

**Fig 33:**
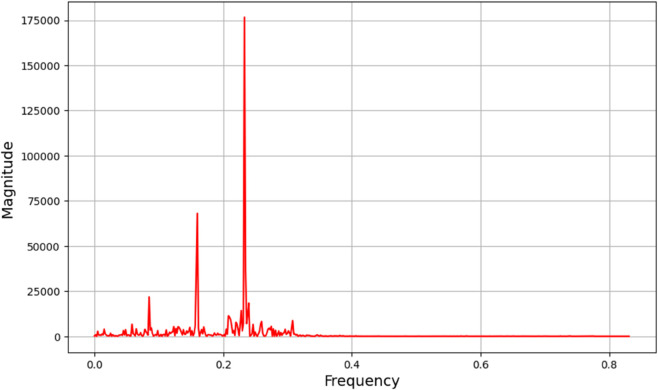
Detection of chaotic behavior through power spectrum for dynamical system (45) with ${𝔉}_{1}=0.85$, ${𝔉}_{2}=-0.45$, and κ=2.28.

**Fig 34:**
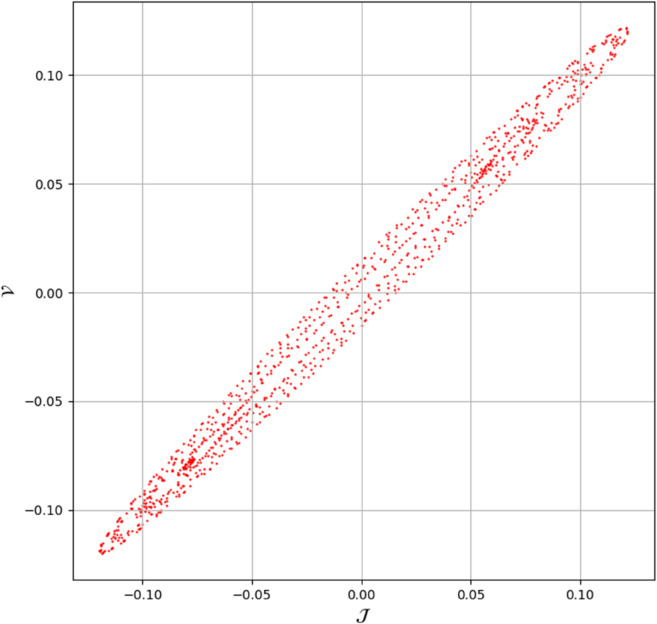
Detection of chaotic behavior through return map for dynamical system (45) with ${𝔉}_{1}=0.85$, ${𝔉}_{2}=-0.45$, and κ=2.28.

**Fig 35:**
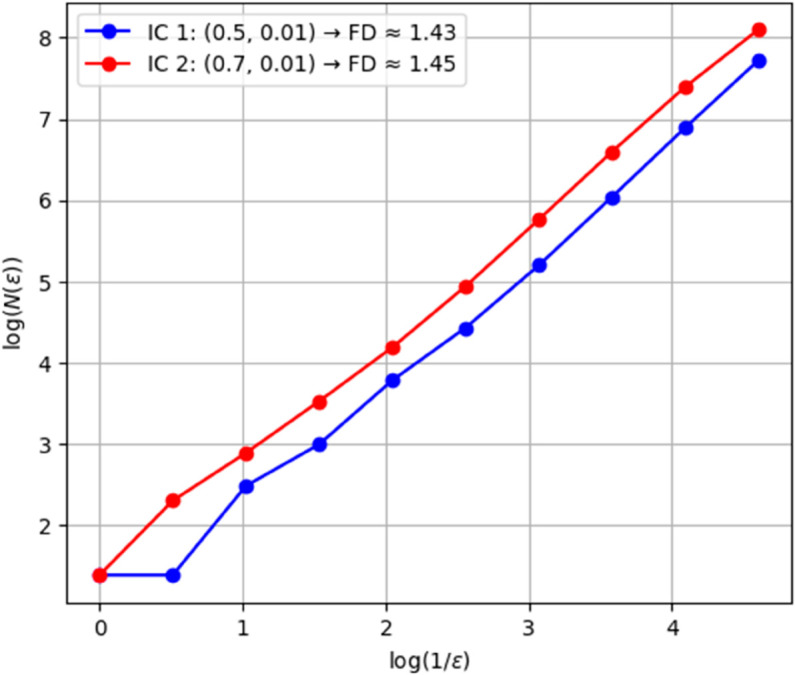
Detection of chaotic behavior through fractal dimension for dynamical system (45) with ${𝔉}_{1}=0.85$, ${𝔉}_{2}=-0.45$, and κ=2.28.

To analyze chaotic dynamics, we will evaluate noise sensitivity using two different approaches [46]. Our investigation centers on the parameters Δ, representing amplitude, and κ, indicating frequency. By varying these parameters, we aim to assess their impact on the system’s chaotic behavior [47,48]. The influence of the applied force on dynamical system (37) is analyzed using various chaos detection methods outlined in the introduction, including phase portraits, Poincaré maps, power spectra, fractal dimensions, return maps, time series, and Lyapunov exponents. These tools are employed to illustrate chaotic behavior in the perturbed system, with graphical representations provided. Identification of chaos via 2D phase portraits for system (45) at ${𝔉}_{1}=0.85$, ${𝔉}_{2}=-0.45$, κ=2.28, and varying Δ behavioue such as:

- When Δ=0.11: The phase portrait demonstrates a complex nested architecture characterized by elaborate loops, signifying quasi-periodic dynamics as shown in Fig 20.
- When Δ=0.33: The trajectory reveals oscillatory behavior accompanied by periodic fluctuations, implying regular dynamical characteristics as shown in Fig 21.
- When Δ=0.44: The heightened complexity manifested in the loops indicates a potential transition towards chaotic dynamics as shown in Fig 22.
- When Δ=0.55: The phase portrait reveals irregular and intersecting trajectories, which serve as a definitive indicator of chaotic motion as shown in Fig 23.
- When Δ=0.66: The structure continues to exhibit intricate features with prominent folding, further substantiating chaotic dynamics as shown in Fig 24.
- When Δ=0.77: The trajectory, characterized by its density and entanglement, corroborates the presence of pronounced chaotic behavior within the system as shown in Fig 25.

- **Time Series:** Figs 26 and 27 presents the time series analysis of system (45) under quasi-periodic feedback, with ${𝔉}_{2}>0$, ${𝔉}_{3}>0$ in shown Fig 26, and ${𝔉}_{2}<0$, ${𝔉}_{3}<0$ in Fig 27.
- **Poincare Maps:** System (45) shows periodic behavior at Δ=0.11, while at Δ=0.33,0.44, and 0.55, it transitions to chaotic behavior as shown in Figs 28–31.
- **Lyapunov Exponents:** The Lyapunov Exponents simulations of the proposed chaotic system indicate one negative and one positive exponent, confirming its chaotic nature, as shown in Fig (32). The computed LEs are *h*_1_ =  + 0.060156, and *h*_2_ = −0.060156. Given the positivity of the first and negativity of the second exponent, the Kaplan-Yorke dimension is determined as follows:
  $$𝔇=\text{ number of Positive Lyapunov Exponents}+\frac{\sum \text{Positive Lyapunov Exponents}}{\left|\text{Negative Lyapunov Exponents}\right|},\;𝔇=1+\frac{\sum \text{ +0.060156}}{\left|\text{- 0.060156}\right|}=2.$$ (46)
  As we can see, 𝔇 is greater than one, which indicates that the system exhibits chaotic behavior. The dynamical system denoted as (45) with parameters $\rho_{1}=0.85$, $\rho_{2}=-0.45$, and κ=2.28 demonstrates chaotic behavior, as substantiated by the diagnostics illustrated in Fig 32.
- **Power Spectrum** The expansive and continuous spectrum reveals a diverse array of excited frequencies, devoid of discrete peaks. This characteristic is indicative of chaotic systems, wherein trajectories exhibit periodicity and a high sensitivity to initial conditions as shown in Fig 33.
- **Return Map:** The plotted points exhibit a fractal-like, irregular configuration as opposed to forming a closed curve or distinct points. This observation corroborates the existence of a strange attractor, which is a defining feature of chaos as shown in Fig 34.
- **Fractal Dimension:** The non-integer value associated with the fractal dimension provides further evidence of chaotic dynamics, as it encapsulates the system’s intricate, self-similar geometry within phase space as shown in Fig 35.

## 7 Conservation laws of Eq (14)

Bluman [49] developed a systematic method for generating important conserved vectors. Their approach entails identifying multiplier ϖ(ϑ,τ,W) of specific orders for a given differential equation, which are subsequently used to determine the associated fluxes:

$$𝔔(\varpi (\vartheta ,\tau ,W)[-S{ℭ}_{0}\frac{\partial^{2}W}{\partial \tau^{2}}+\beta^{2}\frac{\partial^{2}W}{\partial \vartheta^{2}}+{ℜ}_{2}{ℭ}_{0}\frac{\partial }{\partial \tau }(\beta^{2}\frac{\partial^{2}W}{\partial \vartheta^{2}})-{ℜ}_{1}{ℭ}_{0}\frac{\partial W}{\partial \tau }+2\alpha {ℜ}_{1}{ℭ}_{0}W\frac{\partial W}{\partial \tau }])=0.$$ (47)

𝔔 shows the Euler operator:

$$𝔔=\frac{\partial }{\partial W}-D_{\vartheta }\frac{\partial }{\partial W_{\vartheta }}-D_{\tau }\frac{\partial }{\partial W_{\tau }}+D_{\vartheta \vartheta }\frac{\partial }{\partial W_{\vartheta \vartheta }}+D_{\tau \tau }\frac{\partial }{\partial W_{\tau \tau }}+D_{\tau \vartheta }\frac{\partial }{\partial W_{\tau \vartheta }}-.....,$$ (48)

where $D_{\vartheta }$, and $D_{\tau }$ represent the total derivative operators.

$$D_{\vartheta }=\frac{\partial }{\partial \vartheta }+W_{\vartheta }\frac{\partial }{\partial W}+W_{\vartheta \vartheta }\frac{\partial }{\partial W_{\vartheta }}+W_{\vartheta \tau }\frac{\partial }{\partial W_{\tau }}+....\;D_{\tau }=\frac{\partial }{\partial \vartheta }+W_{\tau }\frac{\partial }{\partial W}+W_{\tau \vartheta }\frac{\partial }{\partial W_{\vartheta }}+W_{\tau \tau }\frac{\partial }{\partial W_{\tau }}+.....\cdot$$ (49)

By analyzing Eq (47) and conducting further standard computations, we obtain the following multiplier:

$$\varpi (\vartheta ,\tau ,W)=p_{1}\vartheta +p_{2}.$$ (50)

When $p_{1}=1,p_{2}=0$, the corresponding multiplier is $\varpi^{\left(1\right)}(\vartheta ,\tau ,W)=\vartheta$, yielding the conservation laws:

$$\varpi_{\vartheta }^{\left(1\right)}=C_{0}\alpha R_{1}W^{2}\vartheta +C_{0}\beta^{2}R_{2}\vartheta W_{\vartheta \vartheta }-C_{0}R_{1}W\vartheta -C_{0}SW_{\tau }\vartheta .\;\varpi_{\tau }^{\left(1\right)}=\beta^{2}\vartheta W_{\vartheta }-\beta^{2}W.$$ (51)

When $p_{2}=1,p_{1}=0$, the corresponding multiplier is $\varpi^{\left(2\right)}(\vartheta ,\tau ,W)=1$, yielding the conservation laws :

$$\varpi_{\vartheta }^{\left(2\right)}=C_{0}\alpha R_{1}W^{2}+C_{0}\beta^{2}R_{2}\vartheta W_{\vartheta }-C_{0}R_{1}W-C_{0}SW_{\tau }.\;\varpi_{\tau }^{\left(2\right)}=\beta^{2}W_{\vartheta }.$$ (52)

## 8 Sensitivity analysis of Eq (14)

In this section, we perform sensitivity analyses on the following dynamical system using two distinct initial conditions:

$$\left\{ \begin{array}{c} \frac{d𝒥}{d\varphi }=𝒱,\\ \frac{d𝒱}{d\varphi }={𝔉}_{2}𝒥-{𝔉}_{3}{𝒥}^{2},\\ {𝔉}_{2}=\frac{R_{1}}{R_{2}\beta^{2}a_{2}^{2}},\;\;{𝔉}_{3}=\frac{\alpha R_{1}}{R_{2}\beta^{2}a_{2}^{2}}. \end{array}\right.$$ (53)

**Fig 36:**
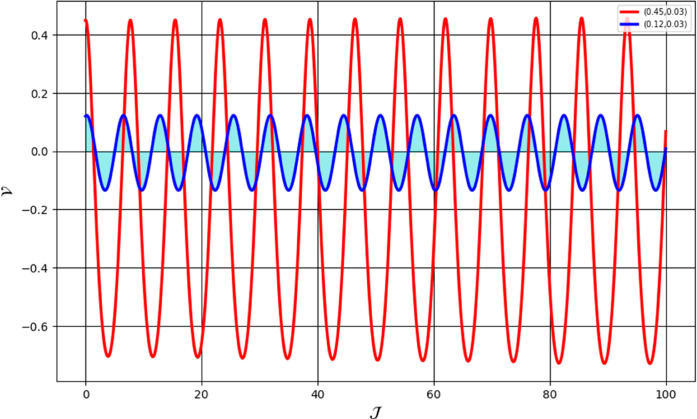
Analysis of sensitivity in the dynamical system (53) using (0.45,0.03) and (0.12,0.03).

**Fig 37:**
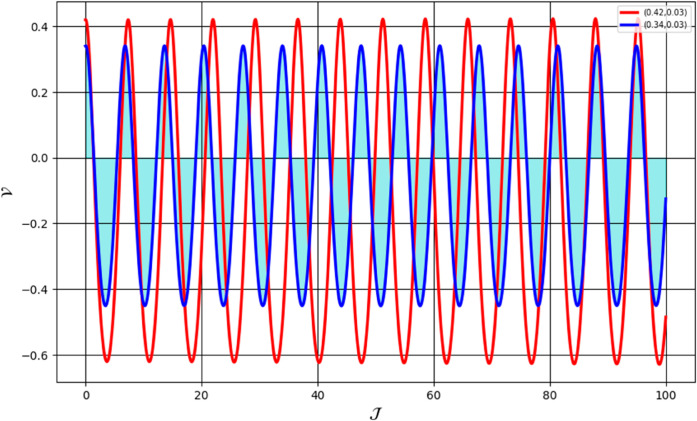
Analysis of sensitivity in the dynamical system (53) using (0.42,0.03) and (0.34,0.03).

**Fig 38:**
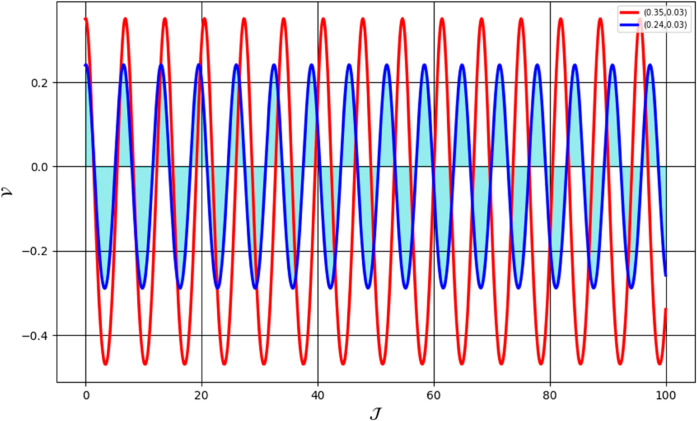
Analysis of sensitivity in the dynamical system (53) using using (0.35,0.03) and (0.24,0.03).

We analyze the system’s behavior across multiple scenarios, each illustrated in distinct figures. Fig 36 presents the sensitivity analysis for the parameter values: β=1.24, *a*_2_ = 0.24, *R*_1_ = 0.38, α=1.56, and *R*_2_ = −0.04. The initial conditions for the red trajectory are set as (𝒥,𝒱)=(0.45,0.03), while for the blue trajectory, they are (𝒥,𝒱))=(0.12,0.03). Fig 37 depicts the sensitivity analysis under different parameter values: β=1.24, *a*_2_ = 0.24, *R*_1_ = 0.38, α=1.56, and *R*_2_ = 0.04. The initial conditions for the red trajectory are (𝒥,𝒱)=(0.35,0.03), whereas for the blue trajectory, they are (𝒥,𝒱)=(0.24,0.03). Fig 38 illustrates the sensitivity analysis for another parameter set: β=1.24, *a*_2_ = −0.24, *R*_1_ = 0.38, α=−1.56, and *R*_2_ = −0.04. The red trajectory starts from (𝒥,𝒱)=(0.42,0.03), while the blue trajectory originates from (𝒥,𝒱)=(0.34,0.03). Notably, even slight variations in the initial parameters result in significant deviations in the system’s behavior, demonstrating a high degree of sensitivity in the examined model.

## 9 Stability analysis of Eq (14)

Examine the perturbed solution expressed as:

$$W(\vartheta ,\tau )=Y_{1}+\Gamma Y_{2}(\vartheta ,\tau ).$$ (54)

It is evident that any constant *Y*_1_ serves as a steady-state solution of Eq (14). Substituting Eq (54) into Eq (14) yields:

$$-SC_{0}\Gamma (Y_{2}{)}_{\tau \tau }+\beta^{2}\Gamma (Y_{2}{)}_{\vartheta \vartheta }+R_{2}C_{0}\beta^{2}\Gamma (Y_{2}{)}_{\vartheta \vartheta \tau }-R_{1}C_{0}\Gamma (Y_{2}{)}_{\tau }+2\alpha R_{1}C_{0}\Gamma (Y_{2}{)}_{\tau }Y_{1}\;+2\alpha R_{1}C_{0}\Gamma^{2}(Y_{2}{)}_{\tau }Y_{2}=0.$$ (55)

Linearizing Eq (55) results in:

$$-SC_{0}\Gamma (Y_{2}{)}_{\tau \tau }+\beta^{2}\Gamma (Y_{2}{)}_{\vartheta \vartheta }+R_{2}C_{0}\beta^{2}\Gamma (Y_{2}{)}_{\vartheta \vartheta \tau }-R_{1}C_{0}\Gamma (Y_{2}{)}_{\tau }=0.$$ (56)

Assume that Eq (56) admits a solution of the form [50]:

$$Y_{1}(\vartheta ,\tau )=e^{ik_{0}\vartheta +{𝔉}_{0}\tau },$$ (57)

where *k*_0_ represents the dimensionless wave number, substituting Eq (57) into Eq (56) and solving for ${𝔉}_{0}$, we obtain:

$${𝔉}_{0}(k_{0})=\frac{-\beta^{2}C_{0}R_{2}K_{0}^{2}-R_{1}C_{0}\mp \sqrt{\beta^{4}C_{0}^{2}R_{2}^{2}K_{0}^{4}+2\beta^{2}C_{0}^{2}R_{1}R_{2}K_{0}^{2}-4S\beta^{2}C_{0}K_{0}^{2}+C_{0}^{2}R_{1}^{2}}}{2SC_{0}}.$$ (58)

According to the stability analysis, the sign of $\text{Re}({𝔉}_{0})$ determines the stability of the system. If $\text{Re}({𝔉}_{0})>0$, the perturbation grows exponentially, indicating an unstable system. If $\text{Re}({𝔉}_{0})<0$, the perturbation decays, leading to a stable system.

## 10 Conclusion

The governing equations of the model are derived using Kirchhoff’s laws applied to the LRC resonant circuit, representing an actin monomer within a filament. By taking the continuum limit, we obtained second-order partial differential equations describing the spatio-temporal voltage distribution. This study aims to conduct the follwong results:

- First, we perform the Lie point symmetry analysis of the considered model and obtain a two-dimensional Lie algebra. It is observed that these Lie symmetries preserve an abelian algebraic structure. Using the identified symmetries, we construct the corresponding symmetry group.
- Next, we derive exact solutions through symmetry reduction by combining the symmetries to reduce the original PDE into an ODE. The tanh method is applied to obtain the soliton solution. To visualize these solutions, we utilized Mathematica to generate 3D and 2D plots as shown in Figs 4–7, illustrating kink and anti-kink solitons. The tanh method simplifies nonlinear PDEs into solvable algebraic forms, making it computationally efficient. It is widely applicable for constructing exact soliton and wave solutions in nonlinear systems.
- Further, we apply the Galilean transformation to the ODE and convert it into a dynamical system. The resulting system is analyzed using bifurcation analysis. We discuss all parameter cases where the system exhibits stable and unstable behavior, as illustrated in Figs 8–19. Finally, we derive the Hamiltonian corresponding to the dynamical system.
- We apply an external forcing term to the dynamical system to study its chaotic behavior. This chaotic nature was demonstrated through time series plots, phase portraits, return maps, power spectrum, Poincaré maps, fractal dimensions, and Lyapunov exponents, as shown in Figs 20–35.
- Sensitivity analysis was carried out using the Runge-Kutta method, as depicted in Figs 36–38.
- Additionally, conservation laws were derived through the multiplier method.
- Finally, a stability analysis was performed, indicating that the system remains stable when $Re({𝔉}_{0})<0$; otherwise, it becomes unstable.

The limitation of bifurcation and chaos analysis lies in their current applicability mainly to second-order ODEs and integrable systems. In future work, this model can be explored numerically using advanced techniques such as the Finite Element Method, Finite Volume Method, Spectral Method, Lattice Boltzmann Method, Pseudo-Spectral Method, and the Lump solution approach. These directions open up possibilities for extending the analysis to more complex nonlinear PDEs, offering a broader scope for future research.

## References

[1] Li L, Cheng B, Dai Z. Novel evolutionary behaviors of N-soliton solutions for the (3 1)-dimensional generalized Camassa–Holm–Kadomtsev–Petciashvili equation. Nonlinear Dynamics. 2024;112(3):2157–73.

[2] Liu HD, Tian B, Feng SP, Chen YQ, Zhou TY. Integrability, bilinearization, Bäcklund transformations and solutions for a generalized variable-coefficient Gardner equation with an external-force term in a fluid or plasma. Nonlinear Dynamics. 2024;112(14):12345–59.

[3] Yang H, Zhang X, Hong Y. Classification, production and carbon stock of harvested wood products in China from 1961 to 2012. BioResources. 2014;9(3):4311–22.

[4] Zhu BP, Wu DW, Zhou QF, Shi J, Shung KK. Lead zirconate titanate thick film with enhanced electrical properties for high frequency transducer applications. Applied Physics Letters. 2008;93(1). 10.1063/1.3095504 PMC2682757 19529788

[5] Muhammad J, Younas U, Hussain E, Ali Q, Sediqmal M, Kedzia K, et al. Solitary wave solutions and sensitivity analysis to the space-time *β*-fractional Pochhammer-Chree equation in elastic medium. Sci Rep. 2024;14(1):28383. doi: 10.1038/s41598-024-79102-x 39551828 PMC11570698

[6] Kumar A, Kumar S, Bohra N, Pillai G, Kapoor R, Rao J. Exploring soliton solutions and interesting wave-form patterns of the (1 1)-dimensional longitudinal wave equation in a magnetic-electro-elastic circular rod. Optical and Quantum Electronics. 2024;56(6):1029.

[7] Kumar S, Kumar A. Lie symmetry reductions and group invariant solutions of (2 1)-dimensional modified Veronese web equation. Nonlinear Dynamics. 2019;98(3):1891–903.

[8] Kumar S, Kumar A, Kharbanda H. Lie symmetry analysis and generalized invariant solutions of (2 1)-dimensional dispersive long wave (DLW) equations. Physica Scripta. 2020;95(6):065207. doi: 10.1088/1402-4896/ab7c3b

[9] Kumar D, Saharan A, Kumar A. Exploring soliton patterns and dynamical analysis of the solitary wave form solutions of the (3 1)-dimensional Wazwaz–Benjamin–Bona–Mahony equation. Modern Physics Letters B. 2025:2550102.

[10] Yusuf A, Sulaiman TA, Abdeljabbar A, Alquran M. Breather waves, analytical solutions and conservation laws using Lie–Backlund symmetries to the (2 1)-dimensional Chaffee–Infante equation. Journal of Ocean Engineering and Science. 2023;8(2):145–51.

[11] Zhiquan Y, Yingyan Z, Shanguang Q, Kepeng H. Types and space distribution characteristics of debris flow disasters along China-Pakistan Highway. Electron J Geotech Eng. 2016;21:191–200.

[12] Sui X, Bai L, Chen Q, Gu G. Influencing factors of microscanning performance based on flat optical component. Chinese Optics Letters. 2011;9(5):052302.

[13] Zhao L, Weng W, Ni M, Shen H, Zhang S, Chen Y, et al. Rubidium salt can effectively relieve the symptoms of DSS-induced ulcerative colitis. Biomedicine & Pharmacotherapy. 2024;181:117574. 39520912 10.1016/j.biopha.2024.117574

[14] Zhang Z, Lin M, Li D, Wu R, Lin R, Yang C. An AUV-enabled dockable platform for long-term dynamic and static monitoring of marine pastures. IEEE Journal of Oceanic Engineering. 2024.

[15] Sui X, Chen Q, Gu G, Liu N. Multi-sampling and filtering technology of IRFPA. Optik. 2011;122(12):1037–41.

[16] Xiao Y, Yang Y, Ye D, Zhang J. Quantitative precision second-order temporal transformation based pose control for spacecraft proximity operations. IEEE Transactions on Aerospace and Electronic Systems. 2024.

[17] Xiao Y, Yang Y, Ye D. Scaling-transformation based attitude tracking control for rigid spacecraft with prescribed time and prescribed bound. IEEE Transactions on Aerospace and Electronic Systems. 2024.

[18] Yang ZQ, Hou KP, Guo TT. Study on the effects of different water-cement ratios on the flow pattern properties of cement grouts. Applied Mechanics and Materials. 2011;71:1264–7.

[19] Yang H, Yuan T, Zhang X, Li S. A decade trend of total factor productivity of key state-owned forestry enterprises in China. Forests. 2016;7(5):97.

[20] Tuszyński JA, Portet S, Dixon JM, Luxford C, Cantiello HF. Ionic wave propagation along actin filaments. Biophys J. 2004;86(4):1890–903. doi: 10.1016/S0006-3495(04)74255-1 15041636 PMC1304047

[21] Faridi WA, Wazwaz AM, Mostafa AM, Myrzakulov R, Umurzakhova Z. The Lie point symmetry criteria and formation of exact analytical solutions for Kairat-II equation: Paul-Painlevé approach. Chaos, Solitons & Fractals. 2024;182:114745.

[22] Arrigo DJ. Symmetry analysis of differential equations: an introduction. John Wiley & Sons; 2015.

[23] Faridi WA, AlQahtani SA. The formation of invariant exact optical soliton solutions of Landau-Ginzburg-Higgs equation via Khater analytical approach. International Journal of Theoretical Physics. 2024;63(2):31.

[24] KopcÂ¸asız B, YaÂ¸sar E. Dual-mode nonlinear Schrödinger equation (DMNLSE): Lie group analysis, group invariant solutions, and conservation laws. International Journal of Modern Physics B. 2024;38(02):2450020.

[25] KopÂ¸casız B, YaÂ¸sar E. *μ*-symmetries and *μ*-conservation laws for the nonlinear dispersive modified Benjamin-Bona-Mahony equation. Journal of Mathematical Sciences and Modelling. 2023;6(3):87–96.

[26] Noether E. Invariant variation problems. Transport Theor Stat Phys. 1971;1(3):186–207.

[27] Ibragimov NH. A new conservation theorem. Journal of Mathematical Analysis and Applications. 2007;333(1):311–28.

[28] Mahmood T, Alhawael G, Akram S, ur Rahman M. Exploring the Lie symmetries, conservation laws, bifurcation analysis and dynamical waveform patterns of diverse exact solution to the Klein–Gordan equation. Optical and Quantum Electronics. 2024;56(12):1978.

[29] Alquran M, Al-deiakeh R. Lie–Backlund symmetry generators and a variety of novel periodic-soliton solutions to the complex-mode of modified Korteweg-de Vries equation. Qualitative Theory of Dynamical Systems. 2024;23(2):95.

[30] Al-Deiakeh R, Alquran M, Ali M, Yusuf A, Momani S. On group of Lie symmetry analysis, explicit series solutions and conservation laws for the time-fractional (2 1)-dimensional Zakharov-Kuznetsov (q, p, r) equation. Journal of Geometry and Physics. 2022;176:104512.

[31] Kumar S, Ma WX, Kumar A. Lie symmetries, optimal system and group-invariant solutions of the (3 1)-dimensional generalized KP equation. Chinese Journal of Physics. 2021;69:1–23.

[32] Kumar S, Kumar D, Kumar A. Lie symmetry analysis for obtaining the abundant exact solutions, optimal system and dynamics of solitons for a higher-dimensional Fokas equation. Chaos, Solitons & Fractals. 2021;142:110507.

[33] Kaveh A. Optimal analysis of structures by concepts of symmetry and regularity. New York: Springer; 2013.

[34] Jhangeer A, Ansari AR, Imran M, Beenish, Riaz MB. Lie symmetry analysis, and traveling wave patterns arising the model of transmission lines. AIMS Mathematics. 2024;9(7):18013–33.

[35] Yang SX, Liu B, Tang M, Yang J, Kuang Y, Zhang MZ, et al. Extraction of flavonoids from Cyclocarya paliurus (Juglandaceae) leaves using ethanol/salt aqueous two-phase system coupled with ultrasonic. Journal of Food Processing and Preservation. 2020;44(6):e14469. doi: 10.1111/jfpp.14469

[36] Huang G, Liang J, Chen X, Lin J, Wei J, Huang D, et al. Isolation and identification of chemical constituents from zhideke granules by ultra-performance liquid chromatography coupled with mass spectrometry. J Anal Methods Chem. 2020;2020:8889607. doi: 10.1155/2020/8889607 33457039 PMC7785344

[37] Sui X, Zeng J, Chen Q, Gu G. High spatial resolution recording of near-infrared hologram based on photo-induced phase transition of vanadium dioxide film. Opt Lett. 2015;40(7):1595–8. doi: 10.1364/OL.40.001595 25831393

[38] Xiu-Bao S, Qian C, Guo-Hua G, Ning L. Research on the response model of microbolometer. Chinese Physics B. 2010;19(10):108702.

[39] Shi D, Li Z. New soliton solutions of the conformable time fractional Drinfel’d–Sokolov–Wilson equation based on the complete discriminant system method. Open Physics. 2024;22(1):20240099.

[40] Beenish SM. Bifurcation, multistability, and soliton dynamics in the stochastic potential Korteweg-de Vries equation. International Journal of Theoretical Physics. 2025;64(5):1–22.

[41] Beenish SM. Exploring quasi-periodic behavior, bifurcation, and traveling wave solutions in the double-chain DNA model. Chaos, Solitons & Fractals. 2025;192:116052.

[42] Craig W. Hamiltonian dynamical systems and applications. Springer; 2008.

[43] Jhangeer A, Beenish, RË‡Â´ıha L. Symmetry analysis, dynamical behavior, and conservation laws of the dual-mode nonlinear fluid model. Ain Shams Engineering Journal. 2025;16(1):103178.

[44] Beenish SM. Analytical solutions and dynamical insights of the modified Benjamin–Bona–Mahony equation with applications in nonlinear optics. Journal of Applied Mathematics and Computing. 2025:1–25.

[45] Li Z, Hussain E. Qualitative analysis and optical solitons for the (1 1)-dimensional Biswas-Milovic equation with parabolic law and nonlocal nonlinearity. Results in Physics. 2024;56:107304.

[46] KopÂ¸casız B. Qualitative analysis and optical soliton solutions galore: scrutinizing the (2 1)-dimensional complex modified Korteweg–de Vries system. Nonlinear Dynamics. 2024;112(23):21321–41.

[47] Liu C, Li Z. The dynamical behavior analysis and the traveling wave solutions of the stochastic Sasa–Satsuma equation. Qualitative theory of dynamical systems. 2024;23(4):157.

[48] Liu J, Li Z, He L, Liu W. Bifurcation, phase portrait and traveling wave solutions of the coupled fractional Lakshmanan–Porsezian–Daniel equation. Qualitative Theory of Dynamical Systems. 2024;23(2):78.

[49] Bluman G, Anco SC. New conservation laws obtained directly from symmetry action on a known conservation law. Journal of Mathematical Analysis and Applications. 2006;322(1):233–50.

[50] Jhangeer A, Beenish. Ferroelectric frontiers: navigating phase portraits, chaos, multistability and sensitivity in thin-film dynamics. Chaos, Solitons & Fractals. 2024;188:115540.

